# The Mechanism of Inhibition of Pyruvate Formate Lyase by Methacrylate

**DOI:** 10.1021/jacs.3c07256

**Published:** 2023-10-05

**Authors:** Juan Carlos Cáceres, August Dolmatch, Brandon L. Greene

**Affiliations:** † Biomolecular Science and Engineering Program, University of California, Santa Barbara, California 93106, United States; ‡ Department of Chemistry and Biochemistry, University of California, Santa Barbara, California 93106, United States

## Abstract

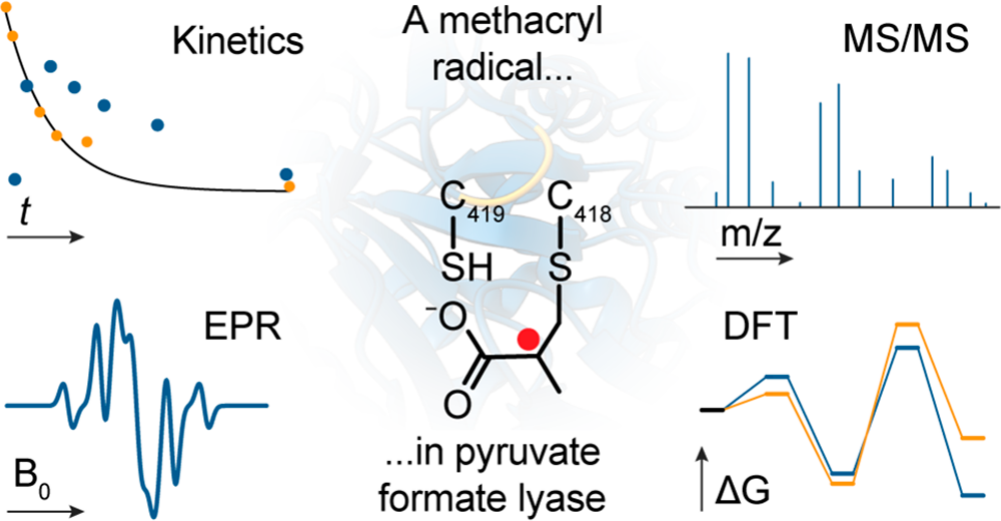

Pyruvate Formate Lyase (PFL) catalyzes acetyl transfer from pyruvate to coenzyme a by a mechanism involving multiple amino acid radicals. A post-translationally installed glycyl radical (G_734_· in *Escherichia coli*) is essential for enzyme activity and two cysteines (C_418_ and C_419_) are proposed to form thiyl radicals during turnover, yet their unique roles in catalysis have not been directly demonstrated with both structural and electronic resolution. Methacrylate is an isostructural analog of pyruvate and an informative irreversible inhibitor of pfl. Here we demonstrate the mechanism of inhibition of pfl by methacrylate. Treatment of activated pfl with methacrylate results in the conversion of the G_734_· to a new radical species, concomitant with enzyme inhibition, centered at *g* = 2.0033. Spectral simulations, reactions with methacrylate isotopologues, and Density Functional Theory (DFT) calculations support our assignment of the radical to a C2 tertiary methacryl radical. The reaction is specific for C_418_, as evidenced by mass spectrometry. The methacryl radical decays over time, reforming G_734_·, and the decay exhibits a H/D solvent isotope effect of 3.4, consistent with H-atom transfer from an ionizable donor, presumably the C_419_ sulfhydryl group. Acrylate also inhibits PFL irreversibly, and alkylates C_418_, but we did not observe an acryl secondary radical in H_2_O or in D_2_O within 10 s, consistent with our DFT calculations and the expected reactivity of a secondary versus tertiary carbon-centered radical. Together, the results support unique roles of the two active site cysteines of PFL and a C_419_ S–H bond dissociation energy between that of a secondary and tertiary C–H bond.

## Introduction

During anaerobic glycolysis in many bacteria, archaea, and some eukaryotes, acetyl-coenzyme A (acetyl-CoA) is produced for substrate-level phosphorylation and biosynthesis by pyruvate formate lyase (PFL), which catalyzes the cleavage of pyruvate to acetyl-CoA and formate.^1−4^ The activity of PFL is dependent on a glycyl radical (G·), post-translationally installed by an S-adenosyl methionine (SAM)-dependent radical activator enzyme (PFL-AE) that abstracts a hydrogen atom from a conserved glycine residue in the C-terminal domain of PFL.^5−8^ An essential G· is the namesake of a diverse class of enzymes, termed glycyl radical enzymes (GREs), where the G· serves as a relatively stable oxidant that transiently generates a cysteine thiyl radical (C·) in the enzyme active site by radical transfer.^9−11^ It is this fleeting C· that catalyzes substrate transformations during turnover. Radical transfer from G· to a pair of cysteines in the active site of PFL is unique among known GREs, and other thiyl radical enzymes such as ribonucleotide reductases, which only contain one substrate activating cysteine.^9,12,13^

**Scheme 1:**
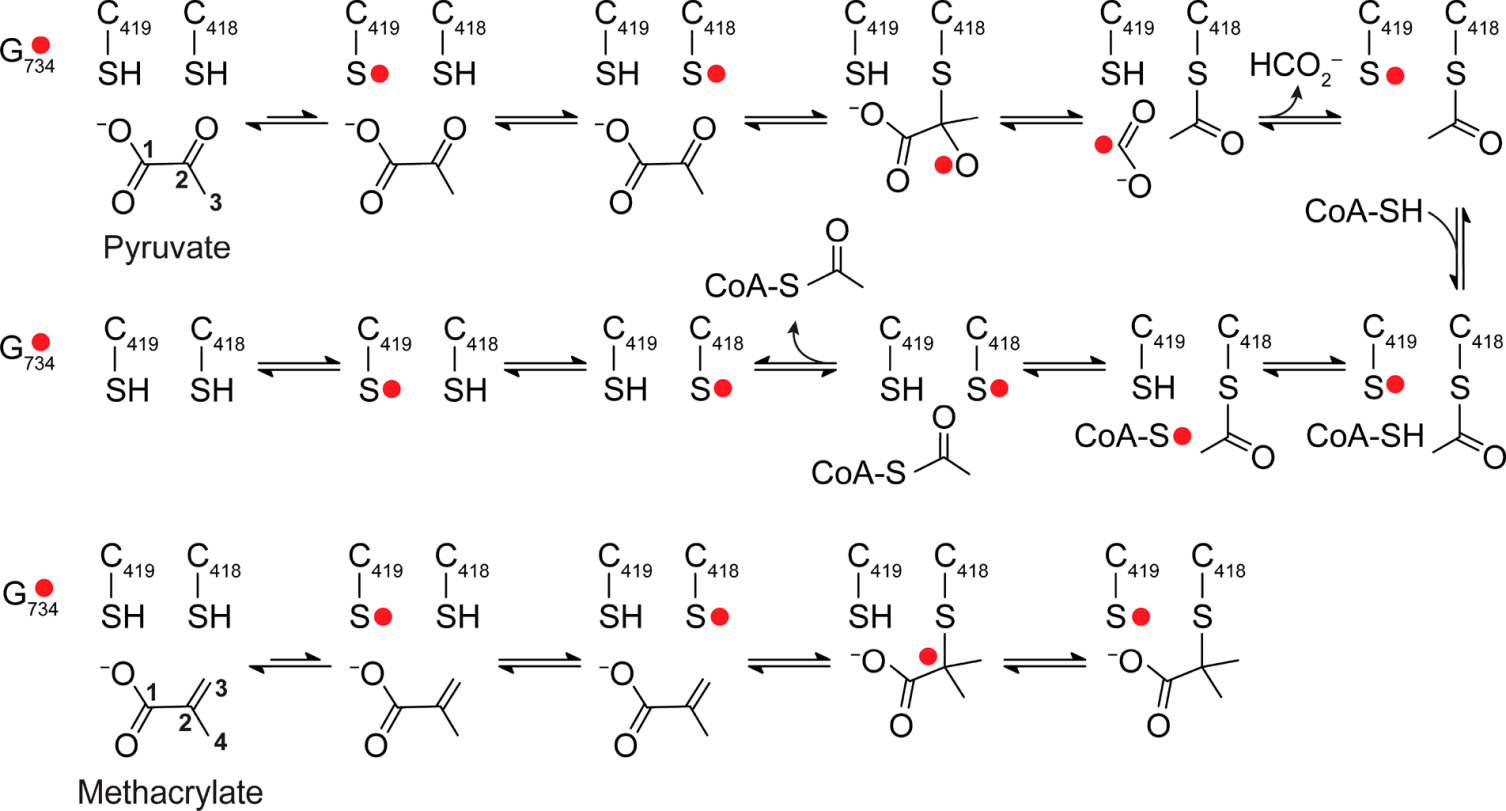
Proposed Reaction Mechanism of PFL with Substrate Pyruvate (top) and Inhibition by Methacrylate (bottom)

As a free amino acid, G· exhibits a reduction potential 110 mV lower than the corresponding C·.^14−19^ Absent radical reduction potential changes due to the protein environment,^20,21^ this difference in reactivity presents an intrinsic challenge to the study of the role of thiyl radicals in GRE catalysis. Consistent with this apparent difference in radical stability, no thiyl radical has been directly observed in the catalytic cycle of PFL, or any other GRE, to our knowledge. The mechanism of PFL and the role of the two cysteines are currently inferred from X-ray crystallographic structures^22−24^ and the effect of mutagenesis,^25,26^ isotopic substitution,^27,28^ and mechanism-based inhibitors^29−34^ on enzyme activity and G· characteristics, focused on the PFL from *Escherichia coli*. A plausible mechanistic proposal is depicted in Scheme 1, which has gained some level of consensus. In this mechanism, radical transfer occurs via a pathway composed of G_734_· ⇌ C_419_ ⇌ C_418_ (*E. coli* numbering) by sequential proton-coupled electron transfer events. The C_418_· then attacks the C2 carbonyl of pyruvate, followed by radical rearrangement and homolysis of the C1–C2 bond of pyruvate to generate an acetylated C_418_ and a CO_2_·^–^ radical anion, that abstracts a H atom from C_419_. Equilibration of C_419_· back to G_734_· completes the ping phase of the ping-pong mechanism.^27^ In the pong phase, H atom abstraction from the CoA sulfhydryl by C_419_·, acetyl exchange, and radical equilibration completes the catalytic cycle. While this mechanistic proposal is consistent with a majority of structural and biochemical observations, the lack of direct observations of radical chemistry with structural specificity leaves ambiguity regarding the unique mechanism and the role of the two cysteines.

Methacrylate is a substrate analog of pyruvate and acts as an irreversible mechanism-based inhibitor of PFL.^34^ It is particularly illuminating as it provides a compelling case for the unique role of the two thiyl radicals during the catalytic cycle. Incubation of active PFL (aPFL) containing the essential G_734_· with 1 mM methacrylate results in complete and irreversible inhibition over 30 min with no apparent effect on the G_734_·.^34^ The product of inhibition is a thioether bond between C_418_ and C3 of methacrylate, which is reduced stereoselectively to a *S*-(2-carboxy-(2*S*)-propyl) adduct.^34^ This biochemical data and the X-ray structure support the mechanism depicted in Scheme 1 and distinct roles for C_418_ and C_419_. Density functional theory (DFT) calculations also support a preference for C_418_· reaction with methacrylate over C_419_· and a slow reduction of the tertiary C2 methacryl radical by the C_419_ sulfhydryl, relative to inactivation.^35^ Unfortunately, these predictions are not supported by direct experimental evidence, limiting the mechanistic insight on the unique role of C_418_ and C_419_.

Here, we report the characterization of the methacryl radical generated upon inhibition of *E. coli* aPFL. Higher concentrations of the inhibitor and faster quenching of the reaction were critical to evidencing the intermediate by X-band electron paramagnetic resonance (EPR) spectroscopy, revealing an inhibitor radical consistent with a tertiary C2 radical of methacrylate. Proteolytic digestion of inhibited PFL and peptide analysis by liquid chromatography-tandem mass spectrometry (LC-MS/MS) resolves a C_418_-methacrylate adduct. Density functional theory (DFT) calculations based on this model and prior studies^35^ reproduce the spectroscopic properties of the C2 radical, and support the stereoselective nature of radical reduction.^34^ Kinetic analysis of the C2 radical decay demonstrates that the reduction of the inhibitor radical is indeed rate determining, as predicted theoretically.^35^ Using a combination of the methacrylate analog, acrylate, solvent kinetic isotope effects (KIE), and DFT calculations we estimate the BDE of the C_419_ thiol S–H bond, placing the first energetic and mechanistic constraint on the H atom transfer chemistry of this residue. The data are consistent with a C_418_· as the radical that adds to C3, and C_419_ as the H atom donor to the C2 radical.

## Materials and Methods

### Materials

DNA oligos were purchased from Integrated DNA Technologies. Electrocompetent DH5α and BL21(DE3) *E. coli*, NEBuilder HiFi DNA Assembly Master Mix, and Phusion polymerase were purchased from New England Biolabs. SapphireAmp Fast PCR-hot-start Master Mix was purchased from Takara Bio. Chloramphenicol and agarose were purchased from Apex Bioresearch Products. LB Miller broth, Terrific Broth (Modified), citrate synthase (porcine heart), malate dehydrogenase (porcine heart), bovine serum albumin, tris(hydroxymethyl)aminomethane base (Tris), β-nicotinamide adenine dinucleotide (NAD^+^), methacrylic acid, sodium acrylate, S-(5′-adenosyl)-l-methionine iodide, KH_2_PO_4_, Triton X-100, glycerol, MgCl_2_·6H_2_O, oxamic acid, sodium pyruvate, CoA, malic acid, iodoacetamide, trifluoroacetic acid, dithiothreitol (DTT), l-cysteine, (NH_4_)Fe^II^(SO_4_)_2_, formic acid, 4-hydroxy-TEMPO, urea, iron ICP-MS standards (TraceCERT), ferrozine (3-(2-Pyridyl)-5,6-diphenyl-1,2,4-triazine-4′,4″-disulfonic acid sodium salt), neocuprine (2,9-Dimethyl-1,10-phenanthroline), ascorbic acid, KMnO_4_, ammonium acetate, phenylmethylsulfonyl fluoride (PMSF), lysozyme from hen egg white, Ribonuclease A from bovine pancreas, Deoxyribonuclease I from bovine pancreas, and Amicon Ultra centrifugal filter units were purchased from Millipore Sigma. We note that Millipore Sigma sodium methacrylate contained variable amounts of an EPR-active species we ascribed to a polymerization inhibitor nitroxide radical. For this reason, methacrylic acid was used with an EPR-silent 4-methoxyphenol polymerization inhibitor. Isopropyl-β-d-1-thioglactopyranoside (IPTG) was purchased from Merck. 5-Deazariboflavin was obtained from Santa Cruz Biotechnologies. Perdeuterated *d*_5_-methacrylacrylic acid was purchased from Polymer Source Inc. DpnI fast digest and Lys-C were purchased from Thermo Scientific. Sequencing grade modified trypsin, and electrocompetent BL21(DE3)pLysS *E. coli* were purchased from Promega Corporation. HiTrap desalting 5 mL columns, and Sephacryl S-100 HR were purchased from Cytiva Life Sciences. Nickel nitrilotriacetic acid (Ni-NTA) agarose resin was purchased from Prometheus Protein Biology Products. Milli-Q water (>17 MΩ) was used for preparing all solutions. The TEVSH plasmid was a gift from Dr. Helena Berglund (Addgene plasmid # 125194).^36^ The plasmid pCAL-n-EK encoding the *pflA* gene was a gift from Prof. Joan Broderick.^37^ The *pflB* gene (Uniprot ID P09373) was codon optimized and synthesized by Integrated DNA Technologies.

### Construction of Plasmids

In order to produce and purify *E. coli* PFL, we cloned the *pflB* gene into a modified pCm1 plasmid (Addgene #174361) using Gibson assembly by PCR.^38,39^ The pCm1 plasmid was modified to encode a 6 × polyhistidine tag (His-tag), followed by an SSG spacer and a TEV protease site N-terminal to the codified protein, and the previous C-terminal tag from pCm1 was removed. We term this new backbone plasmid pCm8. The *pflB* gene was also cloned into pCm8 using Gibson assembly by PCR. A DNA fragment containing *pfl*B gene was amplified by PCR using the primers:

Forward: 5′–tcagggcatgtccgagcttaatgaaaagttagcc–3′

Reverse: 5′–agcctaggttacatagattgagtgaaggtacgagtaataacgt–3′

The primers used to amplify the vector backbone were:

Forward: 5′–tcaatctatgtaacctaggctgctaaacaaagccc–3′

Reverse: 5′–tcggacatgccctgaaaatacaggt–3′

DNA fragments were assembled using NEBuilder HiFi DNA Assembly Master Mix following the manufacturer instructions. Assembly reaction products were transformed into *E. coli* DH5α cells and streaked into chloramphenicol LB-agar plates. Successful transformants were identified using colony PCR with the SapphireAmp fast PCR-hot-start master mix, as directed by the manufacturer, and the fidelity of the cloning was confirmed by Sanger sequencing through UC Berkeley DNA Sequencing Facility using the T7 promoter/terminator and *pflB* specific primers:

Forward: 5′–ggtccggctaccaacgctc–3′

Reverse: 5′–ccaggtcaacagccaggtc–3′

To produce PFL mutants C_418_S and C_419_S we performed site-directed mutagenesis (SDM). For C_418_S we used the following primers:

Forward: 5′–acgatgactacgctattgctagctgcgtaagcc–3′

Reverse: 5′–ggcttacgcagctagcaatagcgtagtcatcgt–3′

For C_419_S we used the following primers:

Forward: 5′–ctacgctattgcttgcagcgtaagcccgatgat–3′

Reverse: 5′–atcatcgggcttacgctgcaagcaatagcgtag–3′

We used Phusion polymerase to perform SDM by PCR per the manufacturer’s instructions and treated the PCR products with DpnI at 37 °C overnight and then transformed them into *E. coli* DH5α electrocompetent cells and streaked the cells on chloramphenicol LB-agar plates. We confirmed the mutagenesis fidelity by Sanger sequencing as described above.

### Protein Expression and Purification

The expression and purification of the TEV protease was performed as previously described.^36^

We expressed wild-type (wt), C_418_S, and C_419_S PFL using the same protocol. BL21(DE3) cells transformed with the corresponding plasmid were inoculated into a 15 mL preculture of LB broth supplemented with 50 μg/mL of chloramphenicol. The preculture was grown overnight at 37 °C in a 50 mL centrifuge tube shaking at 200 r.p.m. The following day, 15 mL of the overnight preculture were inoculated into 1.5 L of terrific broth, supplemented with 50 μg/mL of chloramphenicol, and grown at 37 °C, shaking at 200 r.p.m. Protein expression was induced by adding 0.25 mM IPTG when the OD_600_ reached 1.0, and then the culture was maintained at 25 °C overnight. Cells were harvested by centrifugation at 8,000 × *g* for 10 min, and the collected cell paste, approximately 10–15 g wet cell paste per liter, was flash frozen in liquid N_2_ and stored at −80 °C until purification.

For PLF protein purification we resuspended cell paste in resuspension buffer consisting of 50 mM potassium phosphate (KPi), 150 mM NaCl, 1 mM DTT, and 5% glycerol, adjusted to pH 8.0, and lysed the cells by French press in an Emulsiflex C3 homogenizer at 14,000 psi. Every following step was performed at 4 °C. The cell extract was clarified by centrifugation at 30,000 × *g* for 30 min, and the cell debris was discarded. To remove nucleic acids, 1.5% w/v streptomycin sulfate was added dropwise while stirring and incubated for 10 min before being centrifuged at 30,000 × *g* for 30 min. The pellet was discarded, and the supernatant was filtered through a 0.65 μm filter. The clarified lysate was applied to a 20 mL Ni-NTA column equilibrated with wash buffer composed of 30 mM imidazole, 50 mM KPi, 150 mM NaCl, 1 mM DTT, and 5% glycerol at pH 8.0. The resin was washed with 20 column volumes of wash buffer and eluted with elution buffer in which the imidazole concentration was raised to 400 mM. Fractions containing protein were pooled and concentrated using Amicon Ultra-15 50 kDa molecular weight cutoff (MWCO) centrifugal filter units and then buffer exchanged to resuspension buffer using a HiTrap desalting 5 mL column. To remove the N-terminal His-tag, PFL was incubated with TEV protease in a 1:100 ratio of protease to protein overnight and then the digestion product was injected back into the Ni-NTA column to remove the cleaved N-terminal tag, His-tagged protease, and undigested PFL. The Ni-NTA flow-through was collected and purified, and the untagged protein was concentrated and desalted as described above. The purified protein was stored in resuspension buffer supplemented with 20% w/v glycerol at −80 °C until used.

We modified a previously reported method to express and purify PFL-AE.^40^ BL21(DE3)pLysS cells transformed with pCAL-n-EK-*pflA* were inoculated into a 15 mL preculture of LB broth supplemented with 100 μg/mL of carbenicillin and 35 μg/mL of chloramphenicol. The preculture was grown overnight at 37 °C shaking at 200 r.p.m. 3 mL of cells from the overnight preculture were inoculated into 300 mL of LB broth supplemented with 100 mM KPi buffer pH 7.0, 100 μg/mL of carbenicillin and 35 μg/mL of chloramphenicol and grew at 37 °C with shaking at 200 r.p.m. PFL-AE expression was induced by adding 0.25 mM IPTG, 0.2 mM l-cysteine, and 0.2 mM (NH_4_)Fe^II^(SO_4_)_2_ at OD_600_ of 0.8. The culture was maintained at 30 °C for 5 h, and then l-cysteine and (NH_4_)Fe^II^(SO_4_)_2_ were added to a final concentration of 0.4 mM. The culture was moved to a 4 °C refrigerator and sparged with Argon overnight (15–16 h).

For PFL-AE purification, the culture flasks were moved into a coy glovebox <20 ppm of O_2_ and transferred to centrifuge bottles with an O-ring seal. Cell paste was harvested by centrifugation at 8,000 × g for 5 min. All following steps were performed in the glovebox. Cells were resuspended into anaerobic lysis buffer consisting of 50 mM Tris, 50 mM KCl, 10 mM MgCl_2_, 1% w/v Triton X-100, and 5% w/v glycerol at pH 7.5 in a proportion of 1 mL of buffer per gram of wet cell paste. The resuspended cell pellet slurry was supplemented with 1 mM phenylmethylsulfonyl fluoride, 0.2 mg/mL lysozyme, 1 mM DTT and 10 μg/mL of RNase and DNase, and homogenized on ice, with gentle stirring, for 1 h. The cell slurry was then lysed by ultrasonication with a Qsonica q125 sonicator for 10 min, in cycles of 20 s on–30 s off, on ice. The sample was clarified by centrifugation at 15,000 × *g* for 15 min and filtered using a 0.65 μm filter. The protein extract was then injected into a 70 mL Sephacryl S-100 HR size exclusion column, equilibrated with buffer consisting of 50 mM Tris, 100 mM KCl, and 1 mM DTT at pH 7.5, and resolved at a flow rate of 0.5 mL/min. We collected the dark brown fractions and concentrated them using Amicon Ultra-0.5 10 kDa MWCO centrifugal filter units. The concentrated protein was reinjected in the same column under the same conditions to further improve protein purity. Protein-complexed iron was quantified using the ferrozine assay,^41^ using iron ICP-MS standards (TraceCERT) as an iron standard. Protein concentrations were quantified using the Bradford assay, using bovine serum albumin as a standard.^42^

### PFL Activation

We activated PFL in an anaerobic VAC Atmospheres glovebox (<2 ppm of O_2_) by mixing 50 μM PFL, 5 μM PFL-AE, 2 mM SAM, 20 mM oxamate, and 100 μM 5-deazariboflavin in activation buffer containing 100 mM Tris, 100 mM KCl, 10 mM DTT, and 20% w/v glycerol at pH 7.6 in a 4 mm O.D. EPR tube. We exposed the mixture to a 1 W 405 nm LED light (Thor Laboratories) for 1 h in a thermal bath at 30 °C. The extent of activation was estimated by activity assays and EPR quantitation of the G· (see below).

### PFL Activity Assays

We measured PFL activity spectrophotometrically using a multienzyme assay that couples the PFL-dependent formation of acetyl-CoA to the production of NADH by the oxidation of malate to oxaloacetate and condensation to citrate by malate dehydrogenase and citrate synthase, respectively, as previously reported.^40^ Reactions were initiated by addition of aPFL, and the rate of NADH production was calculated using an extinction coefficient of 6.2 mM^–1^ cm^–1^.^43^ We mixed 5 μL of aPFL at an approximate concentration of 2 μM G·, with 800 μL of the activity assay mix containing 10 mM DTT, 1 mM NAD^+^, 10 mM malate, 2 U/mL citrate synthase, 30 U/mL malate dehydrogenase, and 0.05 mg/mL bovine serum albumin in 100 mM Tris buffer, adjusted to pH 8.1. Reaction initial velocities were determined by detecting NADH production by the absorbance peak at 340 nm, in a VAC Atmospheres glovebox using a custom fiber-coupled Ocean Optics QEPro spectrophotometer and DH-2000-BAL light source.

In order to determine aPFL kinetic parameters, we assayed activity at different concentrations of pyruvate from 0 to 10 mM (120 μM CoA), and 0 to 120 μM for CoA (10 mM pyruvate). In order to calculate specific activities (U/mg, 1 U = 1 μmol/min) we normalized the activity to the measured G· concentration determined by EPR (see below) assuming 1 mol of G· per mole dimer of active PFL, and fitted the calculated initial velocities to a Michaelis–Menten model described by eq 1, using GraphPad Prism version 8.0.0.
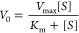
1where *V*_0_ represents the initial velocity, *V*_max_ the maximum velocity, [S] the substrate concentration, and *K*_m_ the Michaelis constant.

For enzyme inactivation kinetics we incubated samples of aPFL with 200 mM methacrylate or acrylate between 0 and 10 min. We took sample aliquots during inactivation and immediately determined the remaining PFL activity as described above. Inhibition time points were performed in duplicate and the average is reported.

### X-Band EPR Spectroscopy

All EPR samples were prepared in a VAC Atmosphere glovebox with <2 ppm of O_2_. To analyze radical intermediates during aPFL inactivation we mixed 180 μL of 50 μM aPFL, in activation buffer, with 20 μL of 2 M methacrylate or acrylate in H_2_O pH adjusted to 7.0. Samples were incubated at room temperature from 10 s to 7 min. The reactions were flash frozen in EPR tubes in liquid nitrogen-cooled isopentane (<−130 °C). EPR spectra of the samples were collected using a Bruker EMXplus EPR spectrometer at 100 K with a frequency of 9.38–9.44 GHz, power of 20 μW, modulation amplitude of 2 G, modulation frequency of 100 kHz, time constant of 0.01 ms, scan time of 20 s, and conversion time of 16 ms. All reported spectra are the average of 30 scans. Spin quantitation was computed from the double integral of the first harmonic signal and referenced to a 4-hydroxy-TEMPO concentration standard. All EPR spectral simulations were performed using EasySpin 6.0.0 software, and confidence intervals and standard deviations of the fitting parameters are reported.^44^

For preparing solvent H/D isotope effect samples, a D_2_O-based buffer was prepared by lyophilizing 100 mM KCl and 100 mM Tris at pH 7.2 and rehydrating the buffer in the same volume of D_2_O. The D_2_O-exchanged solution was measured to be pH* of 7.2, corresponding to a final pD of 7.6 according to eq 2.^45^
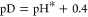
2

To ensure water content <5% in the D_2_O samples, aPFL protein samples were subject to three cycles of 5-fold concentration, followed by 5-fold dilution in D_2_O buffer using 50 kDa MWCO centrifugal filters.

Kinetic constants for the C2· formation and decay in H_2_O and in D_2_O were determined from fitting of the relative G· and C2· contributions to the composite EPR spectra as a function of time in COPASI 4.40. The reaction was fit to an irreversible G· → C2· (*k*_1_) and then C2· → G·* (*k*_2_) model where G·* corresponds to the inhibited PFL G·. The error associated with the fit is reported as the error for the extracted rate constants, and the solvent kinetic isotope effect is reported as *k*_2_(H_2_O)/*k*_2_(D_2_O).

### Peptide Liquid Chromatography-Tandem Mass Spectrometry

We assessed the formation of covalent adducts of methacrylate and acrylate with PFL using tryptic digestion of the protein followed by liquid chromatography tandem mass spectrometry (LC-MS/MS). To digest PFL 100 μg of protein was denatured in 20 μL of 8 M urea in 100 mM ammonium bicarbonate, and 5 mM DTT was added and incubated for 30 min at 37 °C to reduce cysteines. Unreacted cysteines were alkylated with 15 mM iodoacetamide for 30 min in the dark, and the reaction was quenched by adding 20 mM DTT and incubating for 10 min. PFL samples were digested with 0.8 μg Lys-C for 2 h. Next, the digested samples were diluted to <2 M urea using 100 mM ammonium bicarbonate and 0.16 μg of Trypsin were added, and digested at 37 °C overnight. Reactions were stopped by adding formic acid to a final concentration of 1% v/v.

The digested samples (2–5 μg at 0.4–1 μg/μL) were injected into a Waters Acquity H-class Ultra High-Pressure Liquid Chromatography system coupled with a Waters Xevo G2-XS quadrupole time-of-flight (qToF) mass spectrometer. The UPLC stationary phase was a Waters BEH C18 column of 50 mm × 2.1 mm × 1.7 μm and the mobile polar phase a solution of H_2_O with 0.1% v/v formic acid (A) and the apolar mobile phase acetonitrile 0.1% v/v formic acid (B). A solution of 150 pg/μL Leu-enkephalin and 100 fmol/μL Glu-fibrinopeptide B prepared in 25:75 acetonitrile:H_2_O with 0.1% v/v formic acid was used as a lock mass, using the average of 3 scans injected every 30 s. Peptides were resolved in a linear gradient from 20 to 35% B over 30 min with a flow rate of 0.2 mL/min at 60 °C. Eluent from the LC was injected directly into the qToF. The mass spectrometer settings were as follow: source capillary voltage 2.8 kV, sampling cone voltage 20 V, source offset 20 V, source temperature 120 °C, desolvation temperature 350 °C, cone gas flow was 50 L/h and desolvation gas flow was 800 L/h. The spectrometer was set in MS/MS detection mode in positive polarity in the resolution regime, detecting from 50 to 2000 Da, with a scan time of 3 s. Peptides corresponding with the mass of the expected tryptic peptide with the methacrylate, acrylate, or carbamidomethyl modification were fragmented using an energy ramp from 20 to 35 V and a cone voltage of 20 V. The LC-MS/MS data were analyzed using Masslynx.

### Computational Analysis

We obtained coordinate files for the methacryl radical from a previous DFT geometry optimized structure using a small model containing methacrylate and the C_418_–C_419_ dipeptide for EPR spectral parameter predictions.^35^ Rotamers were constructed in ChimeraX by iterative 10° rotation of the methacrylate moiety about the C_418_-bonded C3–C2 bond, and angles are reported relative to the initial *pro*-(*R*) H–C3–C2-C1 dihedral angle of 38°. Single point energies and EPR parameters at each angle were computed for the anionic methacryl radical in Orca 5.0.1^46^ using DFT^47,48^ with the B3LYP^49^ unrestrained hybrid functional and a 6-311++G(d,p) basis set and a conductor-like polarization continuum model^50^ dielectric and index of refraction of chloroform to model the protein environment. Spin contamination was evaluated by the computed expectation value for S^2^, which did not exceed 0.02 above the ideal value of 0.75 for a pure doublet spin system.

Reaction trajectories for methacrylate and acrylate were evaluated in two steps by geometry optimizing reactant and product states and then performing a transition state search. For reactions with methacrylate, the C4 methyl group in the methacrylate structures was replaced by a H and geometry optimized as above. The first step involved the radical Michael addition of C_418_–S· to C3, and we used a small model previously geometry optimized composed of a methyl thiyl radical and methacrylate/acrylate (step 1).^35^ For the second step, methacryl/acryl radical C2· reduction by C_419_–SH (step 2), a second methyl sulfide was included, again starting from a previously geometry optimized state.^35^ All transition states exhibited a single imaginary vibrational frequency, and energies are reported as the sum of Gibbs free energy correction (*G* – *E*_el_) and electronic energy (*E*_el_). To normalize the energy of the overall reaction, the transition state (TS) and product (P) of both step 1 and step 2 were evaluated relative to their respective reactant (R1 or R2), and then the energies of R2, TS2, and P2 were scaled to P1 (P1 = R2).

The kinetic isotope effect of methacryl radical reduction in step 2 was computed using transition state theory by recomputing the Gibbs free energy of activation (Δ*G*^‡^) for the deuterated isotopologue reactant and transition states, assuming the same geometry for the deuterated and protonated reactant and transition states, and calculating *k*_H_/*k*_D_ using eq 3;
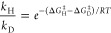
3where *k*_H/D_ are the rate of the corresponding chemical step with either H or D, *R* is the ideal gas constant, and *T* is the temperature in Kelvin. A similar treatment was used to compute the ratio of the rate for inhibitor radical reduction by C_419_ for acrylate (*k*_acryl_) versus methacrylate (*k*_methacryl_).

## Results

### Pyruvate Formate Lyase Inactivation by Methacrylate

To investigate the mechanism of PFL inhibition by methacrylate we recombinantly expressed PFL, including wild-type (wt), C_418_S, and C_419_S variants, as well as PFL-AE in *E. coli*. The PFL proteins were purified by Ni-affinity chromatography and the N-terminal 6 × His tag was subsequently removed, resulting in an overall protein purity of >95% in all cases, based on sodium dodecyl sulfate polyacrylamide gel electrophoresis (Supporting Information Figure S1). The expression yields were 10–20 mg of purified PFL protein per gram of wet cell paste. During the recombinant expression of PFL-AE we applied a continuous sparge of argon to the cell culture after induction to protect the [Fe_4_S_4_] cluster from oxidation. Following anaerobic purification, PFL-AE was estimated to be 60% pure with an overall yield of 10 mg of protein per gram of wet cell paste (Supporting Information Figure S2). Reconstitution of the [Fe_4_S_4_] cluster resulted in PFL-AE iron content of 2.0 ± 0.2 Fe/mol of PFL-AE, likely an underestimate based on the protein impurities.

Treatment of PFL with PFL-AE, in the presence of a photochemical reduction system, and illumination by a 405 nm LED resulted in the accumulation of an asymmetric doublet EPR signal characteristic of the *E. coli* aPFL G_734_· product (Supporting Information Figure S3A).^5^ Under our optimal conditions, PFL-AE was capable of charging 0.5–1.0 G· per PFL dimer of the wt PFL substrate, characteristic of the “half-of-sites” reconstitution and reactivity of the *E. coli* PFL.^28^ The C_418_S and C_419_S PFL variants exhibited similar G· reconstitution efficiency, based on G· EPR signal quantitation (Supporting Information Figure S3B and S3C). We simulated the G· spectrum with an isotropic *g*_iso_ of 2.0037, consistent with prior studies,^51^ and hyperfine coupling (HFC) to a single ^1^H with an isotropic hyperfine coupling constant (A_αCH_) of 40.5 MHz. Coupling to additional nuclei, suggested by prior experimental and theoretical studies,^28,52^ did not impactfully improve the simulation to the experimental data, and were not included in experimental simulations. Transfer of aPFL from H_2_O-based to D_2_O-based buffer results in a narrowing of the doublet signal to an apparent singlet, due to the H/D exchange of the G· and the smaller gyromagnetic ratio of deuterium relative to hydrogen, which is facilitated by the ionizable C_419_ sulfhydryl group (Supporting Information Figure S3D).^26^ The narrowing of the G· spectrum was thus used to confirm H/D exchange of aPFL in subsequent experiments. A table summarizing these, and all subsequent EPR spectral fitting parameters and associated fitting standard deviations are provided in the Supporting Information Table S1.

**Figure 1:**
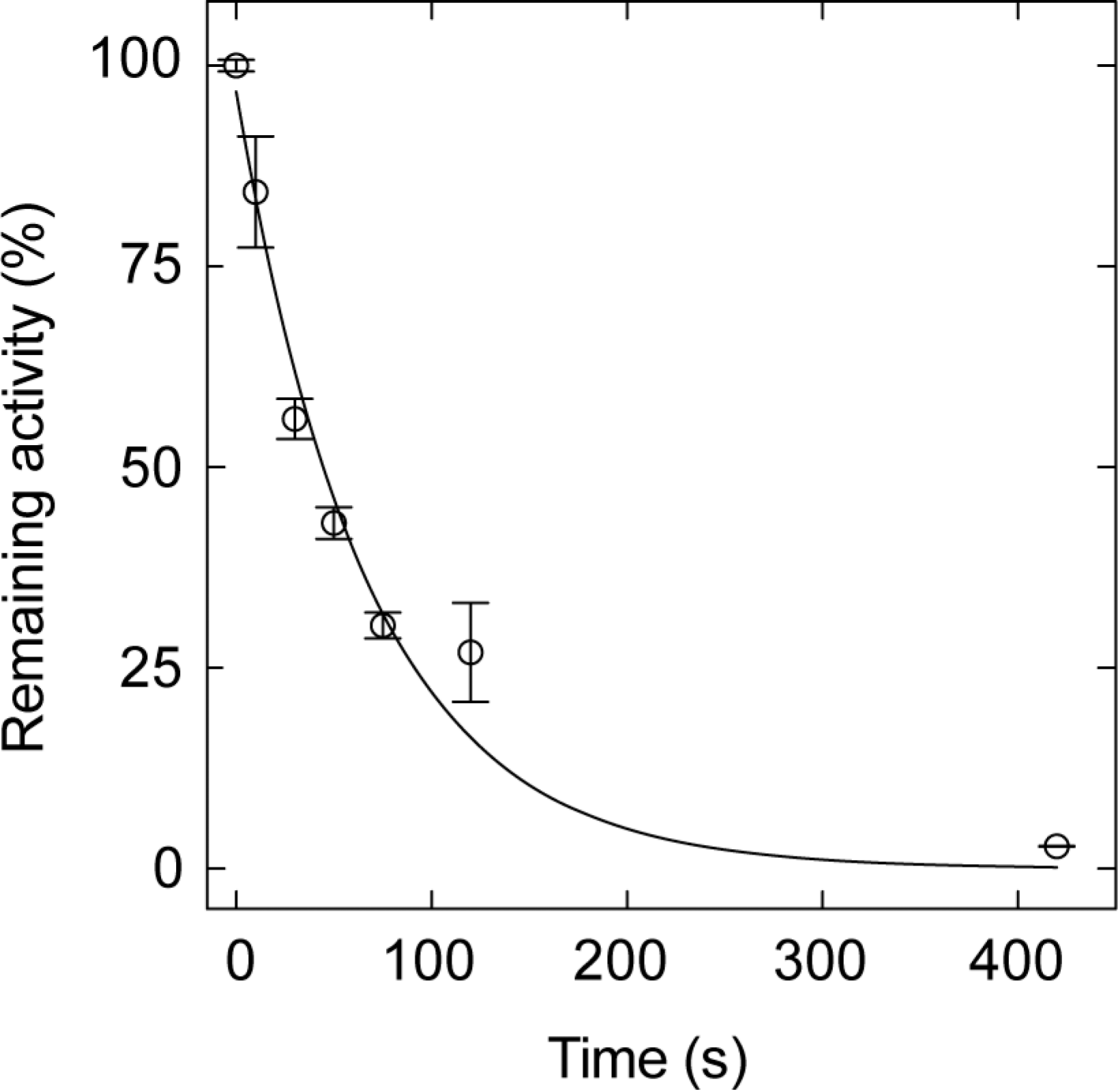
Inhibition of aPFL by methacrylate. aPFL was mixed with 200 mM methacrylate and incubated from 10 to 420 s at 25 °C and aliquots were taken at each time point and assayed for activity by the enzymatic coupled assay. The data were fit to a single exponential decay. Error bars represent the span of two technical replicate experiments.

To evaluate the activity of our recombinantly expressed and activated PFL we employed a multienzyme-coupled spectrophotometric assay.^27^ When normalized to reconstituted active sites, estimated by EPR quantitation of G·, we observed a *k*_cat_ of 225 ± 9 s^–1^ and *K*_m_ of 0.78 ± 0.09 mM for pyruvate and 12 ± 2 μM for CoA at 20 °C, consistent with prior reports of *E. coli* PFL kinetic constants (Supporting Information Figure S4).^27^ The C_418_S and C_419_S variants exhibited activities below the detection limit of the coupled optical assay, which we estimate to be <0.01 s^–1^. Methacrylate is a mechanism-based suicide inhibitor of aPFL with a reported *K*_I_ of 0.4 mM and *k*_inact_ of 0.14 min^–1^.^34^ Indeed, we observe time-dependent inhibition of 50 μM aPFL upon exposure to active site-saturating (200 mM) concentrations of methacrylate, but with an apparent *k*_inact_ of 1 min^–1^ (Figure 1).

### The Methacryl Radical Intermediate

**Figure 2:**
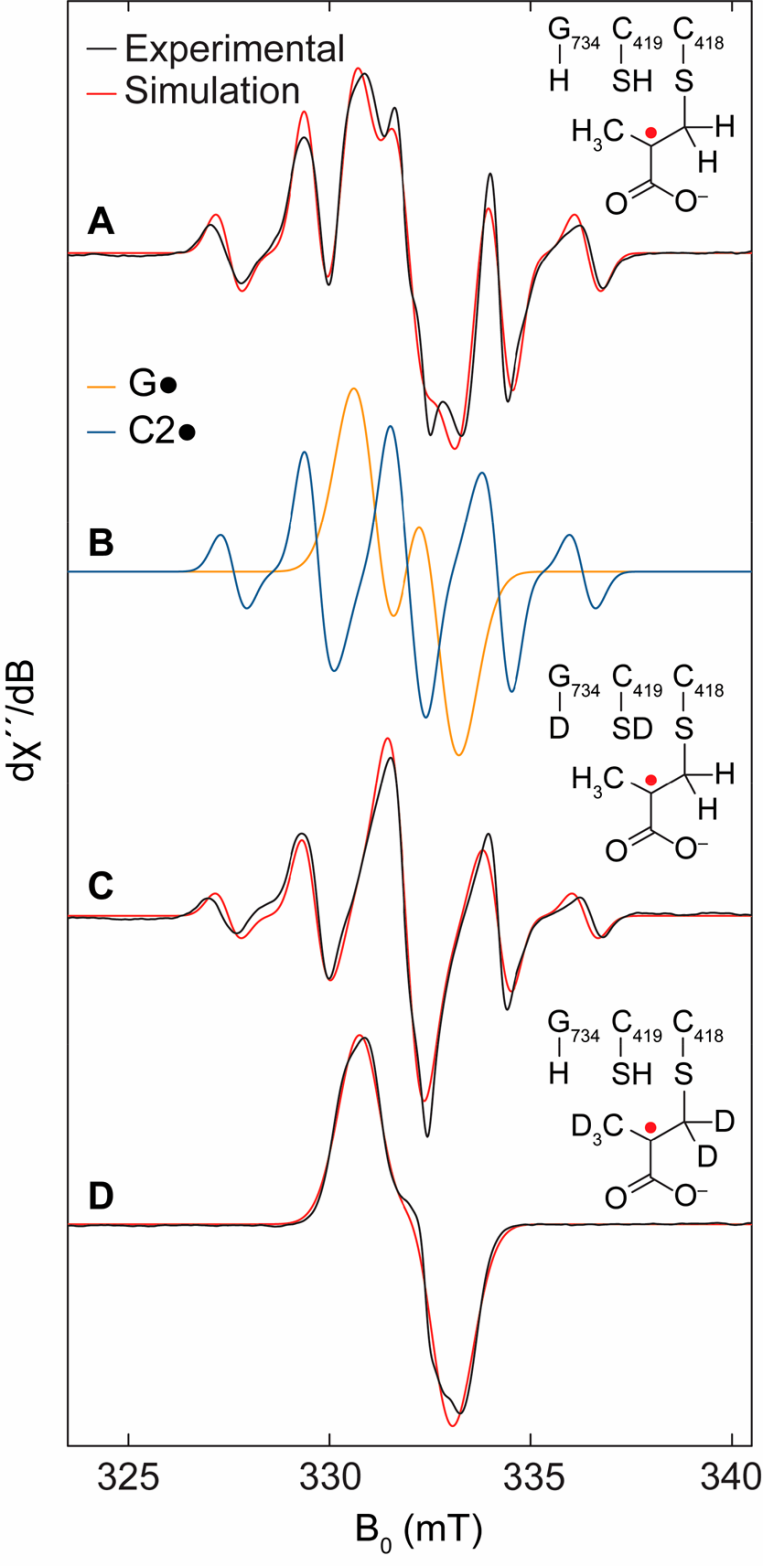
Normalized X-band EPR spectra (black) and simulations (red) of radicals associated with PFL inactivation by methacrylate quenched at 10 s. (A) aPFL reacted with methacrylate in H_2_O. (B) Weighted simulation components assigned to G· (orange) and a methacrylate C2· (blue). (C) aPFL reacted with methacrylate in D_2_O. (D) aPFL reacted with *d*_5_-methacrylate in H_2_O. Experimental spectra were simulated by EasySpin and the simulation spectral parameters are reported in the Supporting Information Table S1. Insets show proposed structures consistent with experimental simulations.

Based on our ability to temporally resolve the inhibition process, we sought to trap a radical intermediate in the inhibition of aPFL. Freeze-quenching the inhibition reaction at 10 s revealed a convoluted multiline EPR spectrum (Figure 2A) composed of G· and a new organic radical species coupled to various spin-active nuclei. We simulated the spectrum using the previously extracted G· parameters, which were held fixed, and allowed the simulation to model a second radical of unknown *g*, as well as the number and strength of potential HFCs to this second radical. Satisfactory simulations included a second radical species comprising 30.9 ± 0.6% of the total EPR intensity, with *g*_iso_ of 2.0033, an isotropic HFC to three identical ^1^H nuclei of 57.9 MHz, and a fourth ^1^H of 69.9 MHz (Figure 2B and Table S1). Inclusion of an additional ^1^H did not statistically improve the simulation fit, as judged by the residuals and the HFC confidence intervals of the additional nuclei. The spectrum is consistent with a C2· methacryl radical, which has been reported previously,^53,54^ but where the radical has minimal electron density overlap with the second methylene ^1^H of C3. No change in the G· signal was observed at any time for C_418_S or C_419_S (Supporting Information Figure S5).

To determine the origin of the hyperfine interactions to the radical intermediate, we repeated the inhibition freeze quenching with aPFL exchanged into D_2_O buffer (Figure 2C). At 10 s the convoluted spectrum exhibits no change to the HFCs of the radical intermediate. The reaction of aPFL with perdeuterated methacrylate in H_2_O, on the other hand, dramatically narrowed the radical intermediate (Figure 2D). These results further support a C2· methacryl radical intermediate assignment.

While our EPR data support a methacryl C2· intermediate, they do not inform on the site of reactivity with aPFL. We turned to peptide analysis of tryptic digestions of PFL by LC-MS/MS. The parental ion of the peptide containing C_418_ and C_419_ eluted at 12.9 min retention time, and the MS/MS fragmentation resolved both y_9_ and y_10_ ions corresponding to the C_419_ (*m*/*z* = 990.5) and C_418_–C_419_ (*m*/*z* = 1150.6) containing peptides respectively, allowing for the unambiguous determination of post-translational modifications at each residue (Supporting Information Figure S6). Following complete inhibition by methacrylate over 10 min, a new peptide is resolved at 14.8 min retention time with y_9_ unchanged, but y_10_ observed at an *m*/*z* of 1179.6, consistent with a methacrylate adduct to C_418_. No adduct is formed with unactivated PFL, confirming the radical nature of the inhibition mechanism, nor in G·-reconstituted C_418_S or C_419_S, suggesting both residues participate in the inactivation process. We also performed the peptide analysis on samples acid-quenched at 10 s, 30 s, and 1 min to terminate radical chemistry at the time of observation of the EPR signal, yielding the same y_10_ ion with the methacrylate adduct with variable intensity (Supporting Information Figure S7).

The methacrylate product observed by LC-MS/MS and EPR is consistent with a tertiary C2· methacryl radical, yet the lack of a fifth hyperfine interaction suggests a specific poise of the radical in the protein active site. To investigate the conformational dependence of the HFC to a putative C2· we performed DFT calculations with an unrestricted hybrid B3LYP functional to simulate the EPR parameters of the radical. As a starting structure, we used a geometry optimized C2· bound to C_418_ through C3 as a C_418_–C_419_ dipeptide.^35^ The dihedral angle between the *pro*-(*R*) methylene H–C3–C2–C1 was systematically rotated about the C3–C2 bond in 10° increments from −180° to +180° relative the initial angle of 38°, and the EPR *g*_iso_ and HFCs were calculated (Supporting Information Figure S8A and Table S2). Two dihedral angle zones with no van der Waals clashes were consistent with the experimental HFCs, −35° and +145°, which show minimal singly occupied molecular orbital (SOMO) overlap between with the *pro*-(*R*) methylene ^1^H (Supporting Information Figure S8B and S8C). The −35° rotamer yields *g*_iso_ of 2.0037 with A_CH3_ of 56 MHz and A_CH2_ of 65 and 3 MHz, whereas the +145° rotamer yields *g*_iso_ of 2.0029 with A_CH3_ of 61 MHz and A_CH2_ of 68 and 2.6 MHz, both in agreement with the experimental spectrum (Supporting Information Table S2). The van der Waals clashes in the other two rotamers may be alleviated by structural dynamics or relaxation, but were not investigated further.

### Methacryl Radical Reactivity

**Figure 3:**
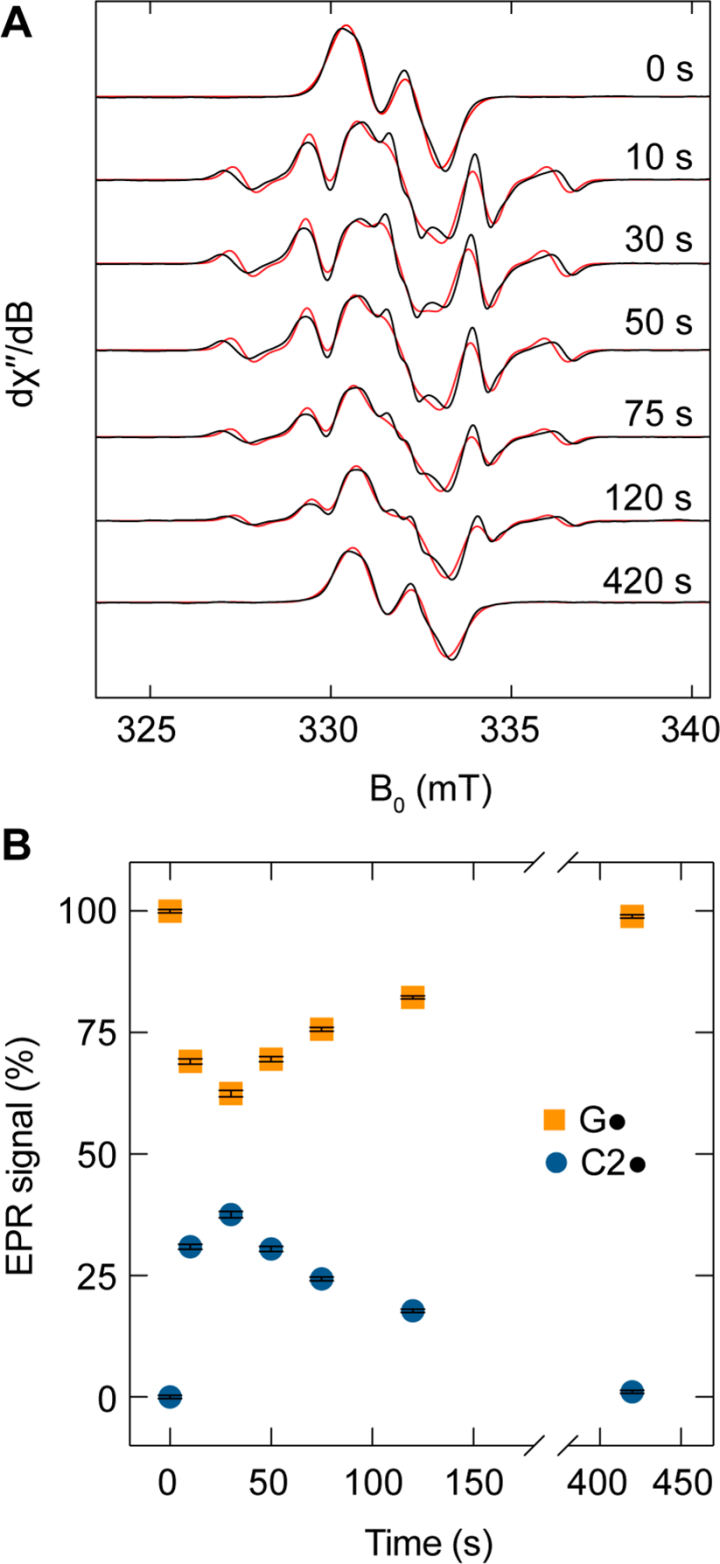
X-band EPR spectra and relative radical content during the inhibition of aPFL by methacrylate. (A) EPR spectra (black) and associated simulations (red) during the aPFL inhibition time course from 0 to 420 s. Experimental EPR spectra were simulated in EasySpin as a convoluted spectrum of G_734_· and C2· of fixed spectral properties determined previously and varying their relative weights. (B) Spectral simulation weights for C2· (blue circles) and G· (orange squares) at each time point. Error bars represent the standard deviation of the fitted covarying weights of the fixed G· and C2· contributions.

The methacryl radical is unstable and decays with concomitant reformation of the G· over the course of minutes at 20 °C. We investigated the time course of C2· formation and decay during the methacrylate inactivation reaction by freeze-quenching reactions of aPFL from 10 s to 7 min (Figure 3) using EPR spectral simulations to quantify G· and C2· over time (Supporting Information Table S3). Using the EPR spectral parameters obtained from Figure 1 we fit the data to a global model to extract the rate constant for inhibition and C2· quenching (Supporting Information Figure S9). The extracted rates were 2.0 ± 0.2 min^–1^ for C2· formation and inhibition, and C2· decay at 1.7 ± 0.1 min^–1^. No other radical species were observed, and the reaction proceeded with <30% total radical loss.

**Figure 4:**
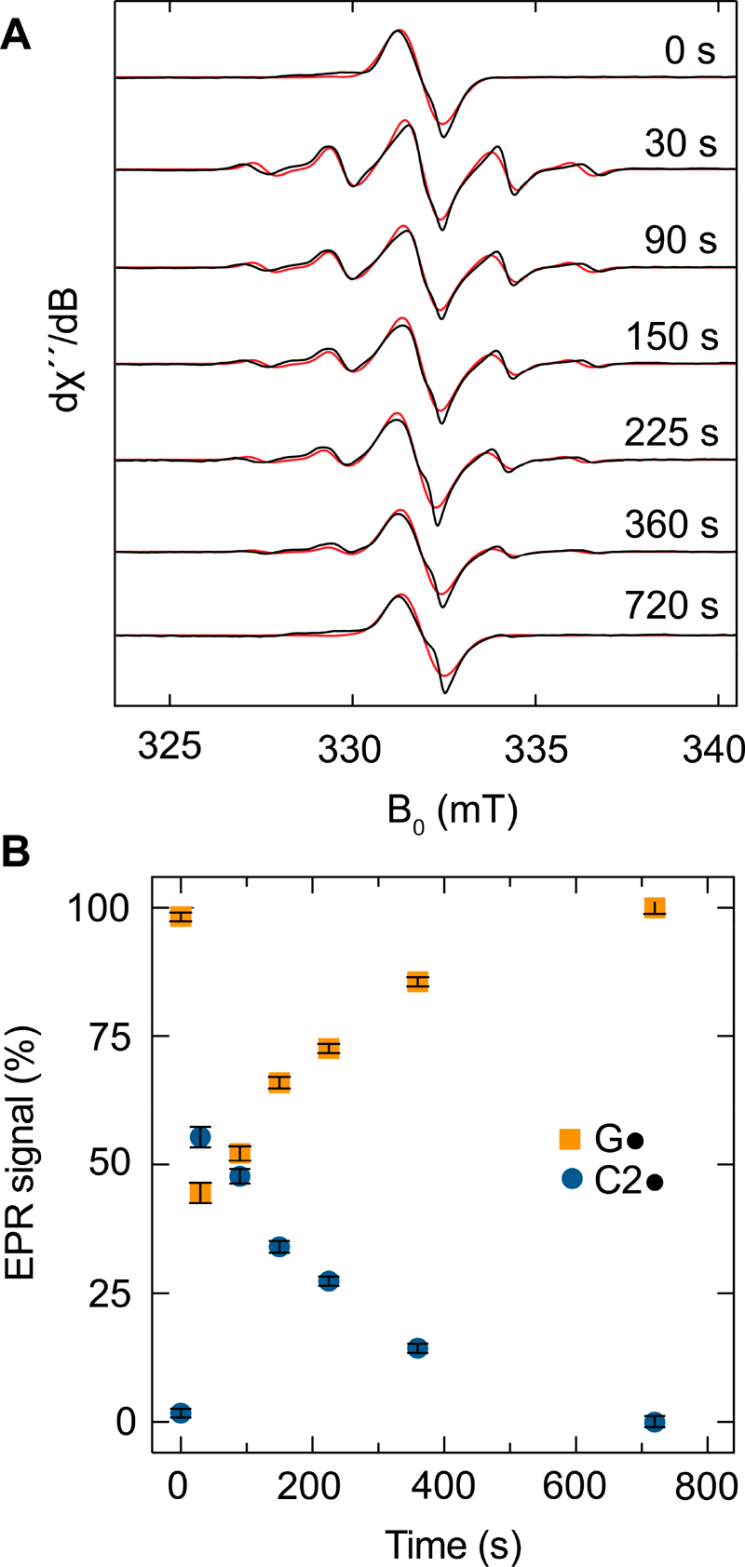
X-band EPR spectra and relative radical content during the inhibition of aPFL by methacrylate. (A) EPR spectra (black) and associated simulations (red) during the aPFL inhibition time course from 0 to 800 s. Experimental EPR spectra were simulated in EasySpin as a convoluted spectrum of (D_2_O) G_734_· and C2· of fixed spectral properties determined previously and varying their relative weights. (B) Spectral simulation weights for C2· (blue circles) and G· (orange squares) at each time point. Error bars represent the standard deviation of the fitted covarying weights of the G_734_· and C2· contributions.

To examine the mechanism of C2· reduction, we repeated the EPR analysis of the methacrylate inhibition process of aPFL in D_2_O buffer (Figure 4). We observed no solvent kinetic isotope effect (KIE) on the formation of the C2· radical (2.0 ± 0.3 min^–1^), but a solvent KIE was observed for the C2· decay and G· reformation (0.50 ± 0.04 min^–1^) of 3.4 ± 0.4, supporting a mechanism of radical reduction by H atom transfer from an ionizable amino acid.

The relatively slow reactivity of the methacryl C2· radical stands in stark juxtaposition to the native reactivity of aPFL, where no radical intermediates have been identified. This could be due to steric constraints of the covalently attached C2·, as opposed to the dissociated CO_2_·^–^ in the native ping phase, or reactivity differences due to BDEs. To shed light on these two potential contributions, we investigated the reactivity of aPFL toward acrylate, which we anticipated to form a much less stable secondary C2· due to steric effects.^55−57^

**Figure 5:**
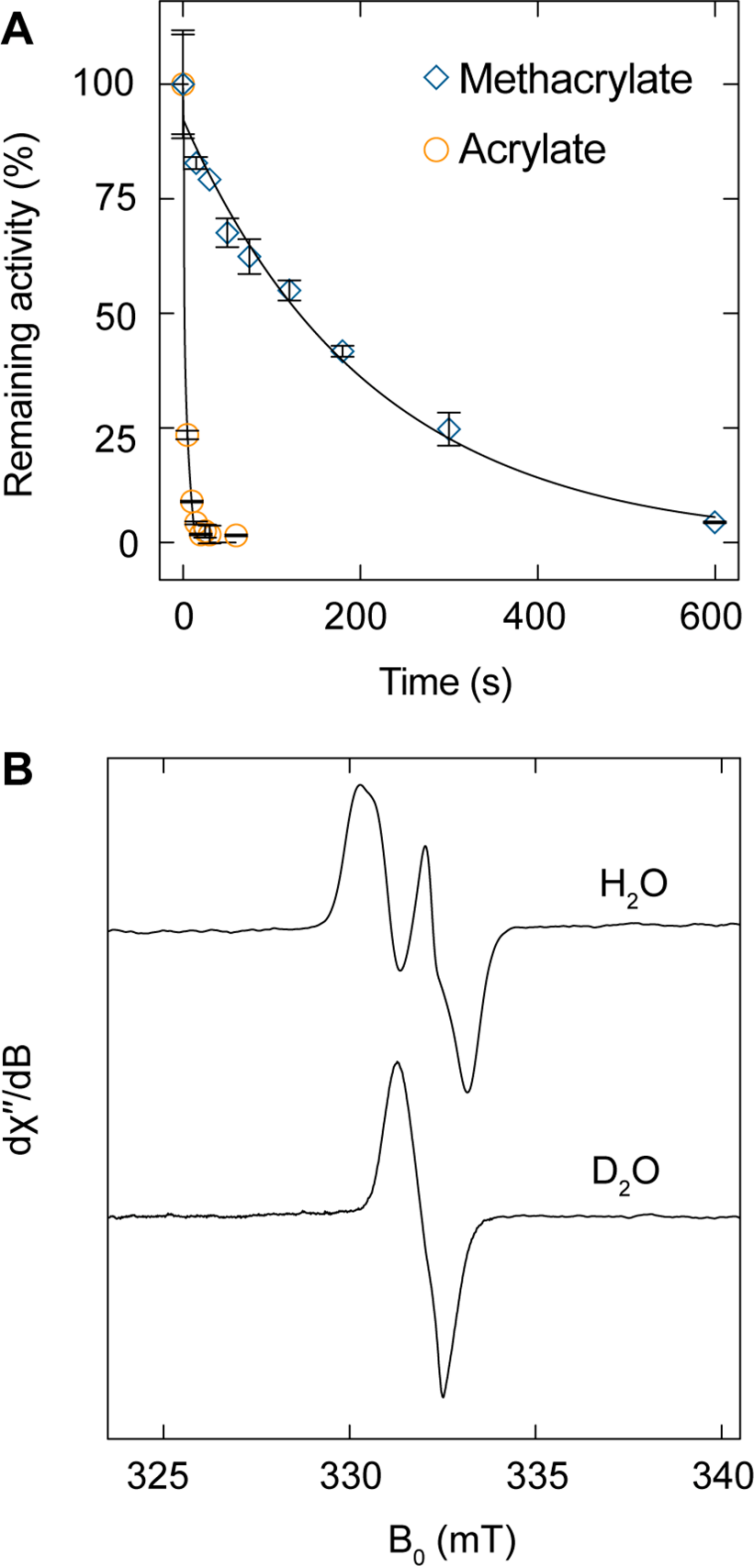
Inhibition of aPFL by acrylate. (A) Remaining activity after inhibition of 5 μM aPFL with 200 mM methacrylate (blue diamonds) or acrylate (orange circles) from 0 to 600 s and fit to a single exponential function (black lines). (B) Normalized X-band EPR spectra reactions of aPFL with acrylate in H_2_O (top) or D_2_O (bottom). Reactions were incubated at room temperature and freeze quenched after 10 s. EPR conditions were: microwave frequency 9.30 GHz, modulation amplitude 2G, at 100 K, 30 scans were averaged for each sample.

We performed kinetic studies to assess acrylate as a PFL suicide inhibitor by detecting PFL remaining activity at different inhibition times. Incubating aPFL with 200 mM acrylate results in activity loss with a higher apparent *k*_inact_ value of >15 min^–1^ relative to that of methacrylate (Figure 5A). The reaction is essentially completed in less than 20 s, which we are unable to accurately characterize due to the nature of the coupled assay and liquid handling. Acrylate adds specifically, and in a radical dependent manner, to C_418_ based on peptide mapping by tryptic digest LC-MS/MS (Supporting Information Figure S10). However, we were not able to capture an EPR spectrum of the acrylate C2· in H_2_O, D_2_O, or in D_2_O at 4 °C at the fastest hand freeze-quenched time accessible of 10 s (Figure 5B).

**Figure 6:**
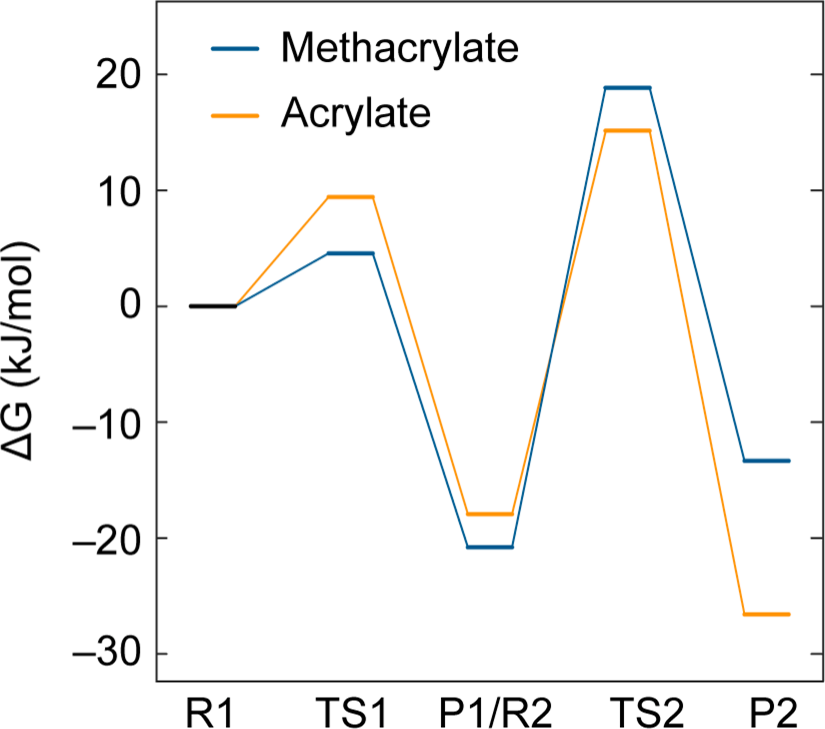
DFT-predicted energy landscape of methacrylate (blue) and acrylate (orange) inhibition of aPFL. Δ*G* is computed relative to the reactant state of step 1 (R1). The mechanism involves two steps, (1) thiyl radical attack at C3, generating a transient C2· followed by (2) H atom transfer from the C_419_ sulfhydryl to C2·. Reactant (R), product (P), and transition states (TS) are indicated.

To gain insight into the relative energetics of the radical chemistry between acrylate and methacrylate, we again turned to DFT. We performed geometry optimizations and transition state searches in a truncated model from a prior study for the reaction with both acrylate and methacrylate and a thiyl radical at C_418_.^35^ The calculated overall energy landscape is shown in Figure 6. The geometry optimized step 1 (thiyl radical addition of C_418_–S· to C3) and step 2 (C2· reduction by C_419_–SH) reactants, transition states, and products are shown in the Supporting Information Figure S11. The thiyl radical addition of C_418_–S· to C3 of either acrylate or methacrylate exhibited low barriers, with Δ*G*^‡^ of 9.4 and 4.6 kJ/mol, respectively, but were both driven to the C2· product with an overall exergonic Δ*G* of −16.7 kJ/mol and −22.1 kJ/mol, respectively. A significant difference was observed between the inhibitors in step 2, where the predicted Δ*G*^‡^ were +33.1 kJ/mol for acrylate and +39.6 kJ/mol for methacrylate. The difference in activation barriers between the two substrates predicts a difference in reactivity of the C2· of acrylate relative to methacrylate (*k*_acryl_/*k*_methacryl_) of 14. For methacrylate, a KIE due to H/D exchange of the C_419_–SH(D) is calculated to be 3.2. The formation of the product of step 2, namely a C_419_–S·, is endergonic for methacrylate by +7.5 kJ/mol, whereas this reaction is exergonic by −8.8 kJ/mol for acrylate, suggesting the C_419_ thiyl radical reactivity is between that the secondary C2· of acrylate and methacrylate.

## Discussion

In the mechanistic study of thiyl radical enzymes, direct observation with structural resolution of radical intermediates associated with the catalytic mechanism is rarely achieved, with the notable exception of ribonucleotide reductase.^58^ For PFL, this has led to some debate about the role of the two active site cysteines during catalysis. Early studies on the mechanism of PFL supported a “thiohemiketal” mechanism of catalysis in which the ping phase was radical-based and the pong phase involved nucleophilic displacement.^25^ Peptide mapping of aPFL reacted with ^14^C2-labeled pyruvate revealed radioactivity associated with C_419_, which was proposed as the site of radical reactivity and acetylation.^30^ The role of C_418_ was suggested by the demonstration that reconstituted C_419_S PFL was charged with an acetyl unit at C_418_ from acetyl-CoA.^25^ The ping and pong phases appeared to be connected by an acyl shift, which was evidenced by reactions of the (C_419_-)acetylated aPFL with hypophosphite, a formate analog, forming a C_418_-ligated acetylphosphonate.^30^ Conversely, the X-ray crystallographic structure of the unactivated PFL bound to pyruvate^23^ and peptide mapping studies with ^14^C2-labeled methacrylate,^34^ supported C_418_ as the site of radical-based substrate activation and acetylation (Scheme 1). This latter mechanism is consistent with the observation of radical equilibration between C_419_ and G_734_ in the absence of catalysis in C_418_S PFL,^26^ and is widely accepted.^9,10^ Neither the thiohemiketal mechanism nor the mechanism described in Scheme 1 are supported by direct radical observation with structural resolution, preventing discernment between the two mechanisms.

By examining the inhibition of aPFL by methacrylate at significantly higher concentrations than those used previously, we have captured a radical intermediate by EPR spectroscopy that is kinetically consistent with inhibition. Based on the radical intermediate EPR signature when generated in H_2_O, D_2_O, and with *d*_5_-methacrylate, we assign this radical intermediate to a C2· tertiary radical of methacrylate, in agreement with previous proposals and theoretical studies.^34,35^ To connect the spectroscopic detection and assignment of the radical intermediate to structure, we characterized the site-specific reactivity of methacrylate for C_418_ by LC-MS/MS, both as an end-point and during the inactivation in acid quenched samples. The acid-quenched samples obviate the potential for acyl shifts that have been reported for PFL with other substrates or inhibitors, and confirm the site-specificity of methacrylate for C_418_. Both EPR and LC-MS/MS studies of the reaction of G·-reconstituted C_419_S showed no evidence of inhibitor radical formation or a methacrylate adduct at C_418_, respectively. These observations support the role of C_419_ as a radical mediator between G_734_· and C_418_. We also gained further insight into the structure through DFT modeling, which supports two rotamers that are consistent with the simulated EPR spectral properties. These two configurations correspond to the orientation of C2· that would generate either 2-(*R*) or 2-(*S*) stereoisomer products, following reduction by H atom transfer. An inspection of the two DFT structures and the X-ray structure of the pyruvate-bound PFL (Supporting Information Figure S12), and the enantioselectivity of the reduction process,^34^ strongly favors the −35° orientation, forming the 2-(*S*) enantiomer, where the vinyl group of methacrylate takes the position of the carbonyl in the pyruvate substrate.

The C2· radical quenching is informative regarding the second half of the ping phase of the reaction mechanism of PFL, where C2· serves as a surrogate for the CO_2_·^–^ in the native reaction. We measured a solvent KIE of 3 for the C2· reduction in H_2_O versus in D_2_O, which confirms that the inhibitor radical is reduced by an ionizable residue capable of H atom transfer. The EPR spectrum of aPFL in D_2_O demonstrates that the C_419_ sulfhydryl H/D exchanges with the solvent, and is the nearest redox-active residue to the substrate in the X-ray structure.^23^ The observed KIE is in agreement with the DFT calculated KIE of 3.2, consistent with the proposed mechanism of C2· reduction by C_419_.

Our observation of a long-lived C2· and DFT analysis is consistent with previous theoretical predictions of a rate determining C2· reduction.^35^ The accumulation of C2· suggests that the tertiary radical is more stable than the corresponding C_419_ thiyl radical, but C2· reduction is ultimately driven forward by radical equilibration back to G_734_·, precluding the direct observation of a transient C_419_ thiyl radical. Using acrylate as a methacrylate analog with a destabilized secondary C2·, we show that this radical is unstable, yet still specific for C_418_. Comparing the product and reactant states of the acrylate reaction by DFT, we estimate that the C_419_ thiol S–H BDE is higher than a tertiary radical and nearly thermoneutral with a carbon secondary radical. Estimates of the bond dissociation energy of the corresponding isobutanoate C2 range from 347 to 355 kJ/mol,^59,60^ and the shift from a tertiary to secondary C2 increases the reactivity by +18 kJ/mol.^60^ Our DFT computed Δ*G* value for C2 reduction of the acryl radical is −8.8 kJ/mol placing the corresponding C_419_ thiol between 356 and 364 kJ/mol, which is in agreement with prior experimental or theoretical studies on the free cysteine amino acid from 353 to 365 kJ/mol.^15,61,62^ These estimates neglect the role of the protein environment, which may significantly alter the radical reduction potentials of the inhibitor or the active site cysteines. The reported formate C–H BDE varies widely, but has been estimated to be 360–380 kJ/mol, making the corresponding step 2 in the native mechanism mildly exergonic.^63−66^

Many thiyl radical enzymes activate secondary carbon centers with high turnover frequencies (*k*_cat_ > 100 s^–1^), including the class II ribonucleotide reductase,^67,68^ PFL,^27^ and 1,2-eliminases (*e.g*., choline trimethylamine-lyase,^69^ propane 1,2-diol dehydrate,^70^ glycerol dehydratase,^71^ 4-hydroxyproline dehydratase,^70^ and isethionate sulfite lyase^72,73^). Interestingly, GREs proposed to activate primary carbon centers, including X-succinate synthases (X = benzyl, 4-isopropylbenzyl, hydroxybenzyl, naphthyl-2-methyl, and 1-methylalkyl)^74,75^ and C–P lyase^76^ exhibit lower turnover rates (*k*_cat_ < 1 s^–1^). Our results may provide a thermodynamic explanation for the kinetic privilege of the thiyl radical enzymes activating secondary, rather than primary carbon centers, although a primary carbon radical was not investigated in this study. This reactivity may also inform mechanistic investigation, where new tertiary centers may stabilize substrate analog radicals sufficiently long enough to be observed and characterized.

## Supporting Information Available

Expression and Purification of wt, C_418_S, and C_419_S PFL, expression and Purification of wt PFL-AE, activation and EPR characterization of aPFL G·, EPR simulation parameters, kinetic characterization of aPFL for pyruvate and CoA, EPR analysis of reactions of methacrylate and C_418_S/C_419_S PFL, peptide LC-MS/MS of aPFL and methacrylate inhibited PFL, DFT predicted HFCs and SOMOs, and comparison of X-ray and DFT predicted structures. (PDF)
