# Supplementary material for: The Mechanism of Inhibition of Pyruvate Formate Lyase by Methacrylate
Source: J Am Chem Soc. 2023 Oct 5;145(41):22504–15. doi: 10.1021/jacs.3c07256 (PMC10591478; doi:10.1021/jacs.3c07256)
Supplement: Supplementary file 1 — ja3c07256_si_001.pdf [file ja3c07256_si_001.pdf]

Supplementary Information

The Mechanism of Inhibition of Pyruvate Formate Lyase by Methacrylate

Juan Carlos Cáceres,[1] August Dolmatch,[2] and Brandon L. Greene[1,2*]

[1] Biomolecular Science and Engineering Program, University of California, Santa Barbara, CA, United States

[2] Department of Chemistry and Biochemistry, University of California, Santa Barbara, CA, United States

*greene@chem.ucsb.edu

TABLE OF CONTENTS

**Content**	**Page**
**Figure S1**. Expression and Purification of wt, $C_{418}S$, and $C_{419}S$ PFL.	3
**Figure S2**. Expression and Purification of wt PFL-AE.	4
**Figure S3**. Activation and EPR characterization of aPFL G●.	5
**Table S1**. EPR simulation parameters for G● and C2●.	6
**Figure S4**. Kinetic characterization of aPFL for pyruvate and CoA.	7
**Figure S5**. $C_{418}S$ and $C_{419}S$ reactivity with methacrylate.	10
**Figure S6**. LC-MS/MS of aPFL peptide of methacrylate inhibited PFL.	11
**Figure S7**. LC-MS/MS of methacrylate inhibition of PFL at 30 s vs. 10 mins.	12
**Figure S8**. DFT predicted HFC constants and SOMOs.	13
**Table S2**. DFT-calculated EPR parameters for rotamers of the C2● intermediate.	14
**Table S3**. Contribution of G_{734}● and C2● for aPFL inhibition by methacrylate.	15
**Table S4**. Contribution of G_{734}● and C2● for aPFL inhibition by methacrylate in D_2O.	16
**Figure S9**. Global kinetic simulations for G●/C2● formation and decay	17
**Figure S10**. LC-MS/MS of aPFL peptide of acrylate inhibited PFL.	18
**Figure S11.** DFT structures for methacrylate and acrylate inhibition intermediates.	19
**Figure S12**. Comparison of X-ray and DFT predicted structures.	20
**References**	21

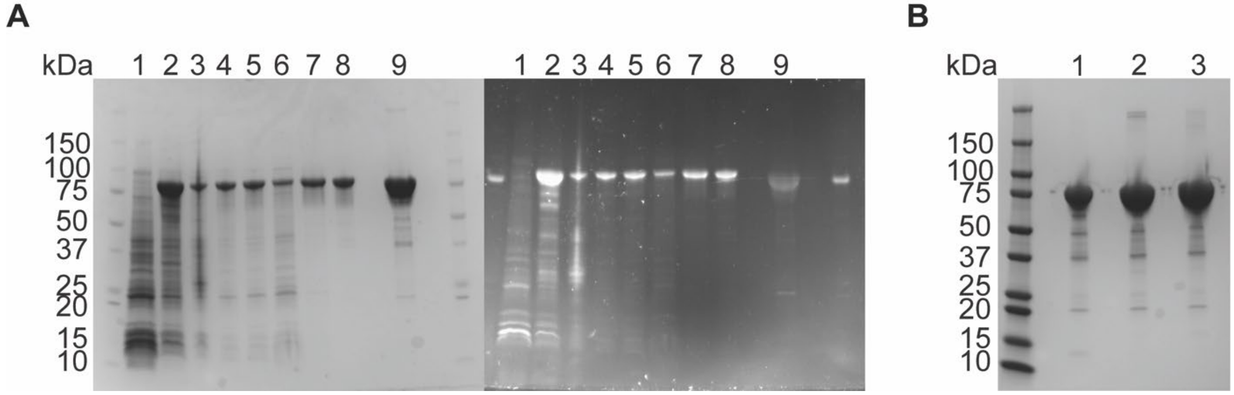

Figure S1. Representative expression of wt PFL and purity of wt, $C_{418}S$, and $C_{419}S$ PFL. **A** Coomassie (left) and InVision™ His-Tag In-Gel (right) stained SDS-PAGE gel of wt PFL expression and purification. Lanes: 1, un-induced whole cells; 2, induced whole cells; 3, cell lysate debris; 4, cell lysate supernatant; 5, streptomycin precipitation supernatant; 6, Ni-NTA column flow-through at 30 mM imidazole; 7, wash with 30 mM imidazole; 8, eluted PFL with 400 mM imidazole; 9, 12.5 µg of Ni-NTA column flow-through of TEV digested PFL. **B** Comparison of purity of 12.5 µg of wt (lane 1), $C_{418}S$ (lane 2), and $C_{419}S$ (lane 3) PFL.

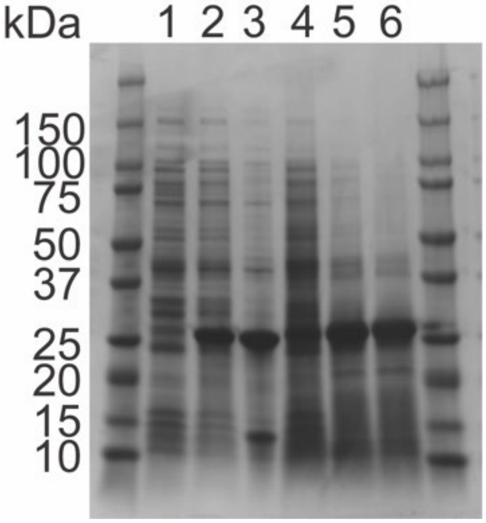

Figure S2. SDS-PAGE analysis of representative PFL-AE purification. Lanes: 1, un-induced whole cells; 2, whole cell induceds; 3, cell lysate debris; 4, cell lysate supernatant; 5, pooled brown fractions from a size exclusion chromatography; 6, pooled brown fractions from the second size exclusion column.

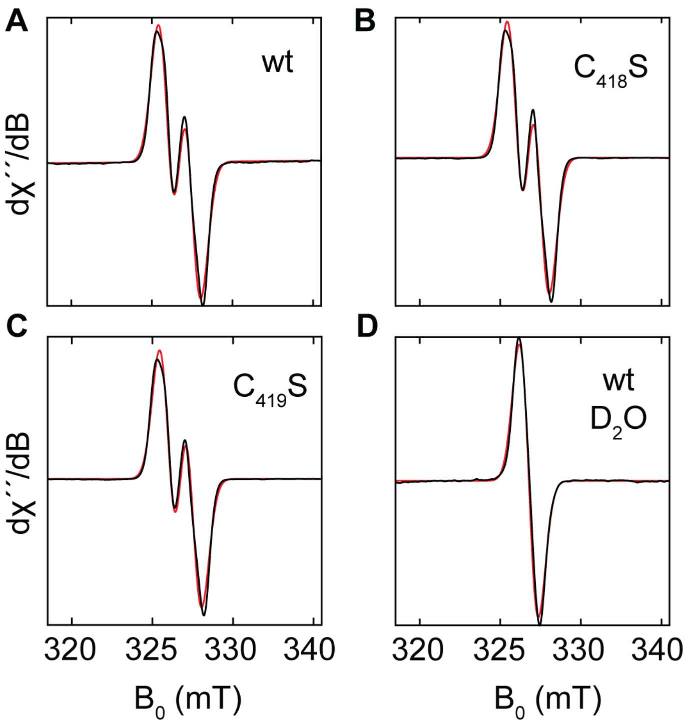

Figure S3. Activation and EPR characterization of aPFL G_{734}●. EPR spectra (black) of G●-reconstituted **A** aPFL wt, **B** PFL C_{418}S, **C** PFL C_{419}S, and **D** aPFL wt, buffer exchanged into D_2O (black trace). EPR spectra were recorded at 100 K, 9.30 GHz, modulation amplitude 2 G, and 30 scans were averaged. Simulations were generated in Easyspin (red trace), *g*-tensors and hyperfine coupling constants are summarized in **Table S1**.

Table S1. EPR simulation parameters for G● and C2●. Errors, reported in parenthesis for the last significant digit, are the standard deviations of the EasySpin simulation parameters.

Species	g_{iso}	$A_{\alpha CH}$ (MHz)	
G_{734}●	2.00365 (3)	40.5 (2)	
G_{734}● (D_2O)	2.00365 (3)	7 (4)	
**Species**	**g_{iso}**	**A_{CH3} (MHz)**	**A_{CH2A} (MHz)**
C2● (H_2O)	2.00331 (2)	57.9 (1)	69.9 (2)
C2● (D_2O)	2.00331 (2)	57.9 (1)	69.9 (2)
C2● (H_2O, d_5-MA)	2.00331 (2)	10 (130)	12 (5)

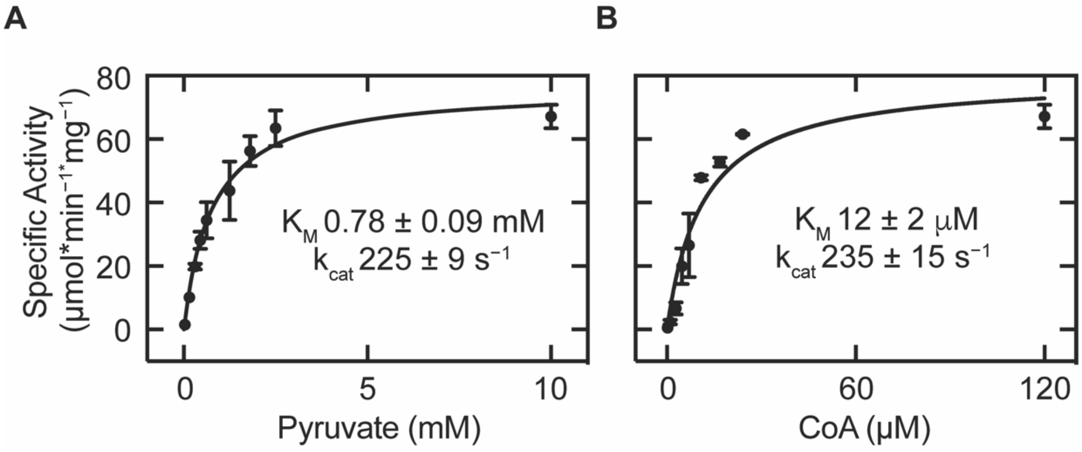

Figure S4. Kinetic characterization of aPFL. **A** Michaelis-Menten plot for aPFL with the substrate pyruvate (120 μM of CoA). **B** Michaelis-Menten plot for aPFL with the substrate CoA (10 mM pyruvate). A Michaelis-Menten kinetic model was fitted and the parameters k_{cat} and K_M were obtained, error bars correspond to the span of 2 technical replicates and kinetic parameters errors correspond to the fit 95% confidence intervals.

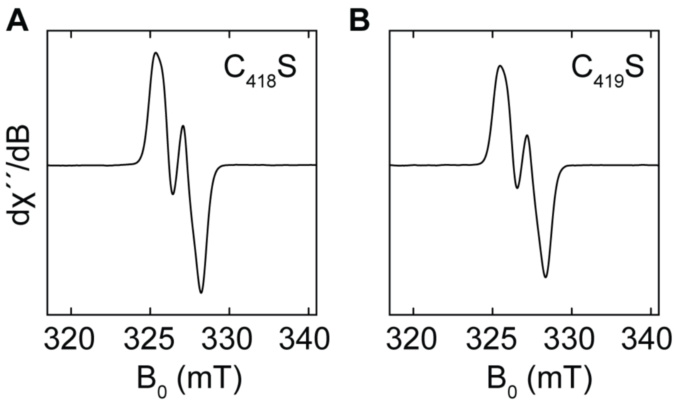

Figure S5. C418S and C419S reactivity with methacrylate. **A** EPR spectra of reconstituted C418S and **B** C419S PFL reacted with 200 mM methacrylate and freeze-quenched after 10 s. Spectra were recorded at 100 K, microwave frequency 9.30 GHz, modulation amplitude 2 G, and 30 scans were averaged.

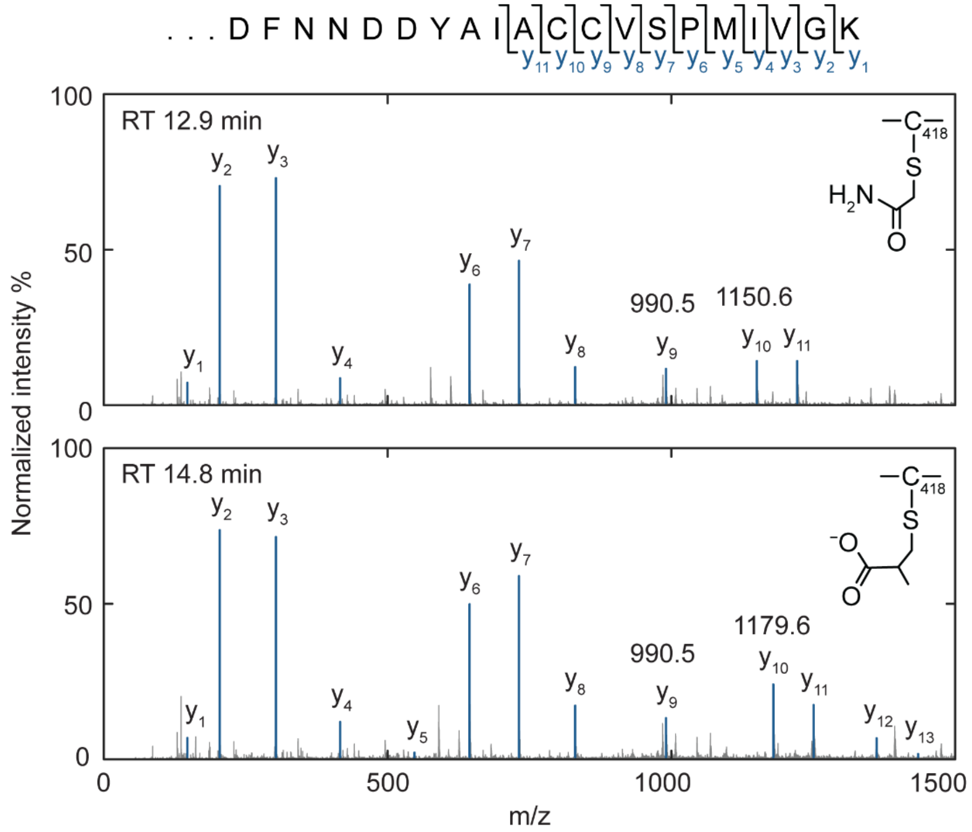

Figure S6. Peptide LC-MS/MS of aPFL inhibited with methacrylate. Wild type aPFL was reacted with 0 (top) or 10 mM (bottom) methacrylate and incubated for 10 minutes at 20 °C. The reaction product was alkylated by iodoacetamide, digested with trypsin, acidified, and analyzed by LC-MS/MS. Fragments of the +4 ion peptide NH_3^+-V_{391}SIDTSSLQYENDDLMRPDFNNDDYAIA$C_{418}C_{419}$VSPMIVG_{426}-CO_2H were detected with a retention time of 12.9 mins and an m/z of 1088.99 for the 0 mM methacrylate sample corresponding to the peptide modified with two iodoacetamide alkylations at C_{419} (y_9 m/z = 990.5 for the +1 ion) and C_{418} (y_{10} m/z = 1050.6 for the +1 ion). Mass spectra were normalized to y_3 at 75%. Chromatograms of the 10 mM methacrylate-reacted PFL revealed a second +4 ion peptide with a retention time of 14.8 mins and m/z of 1095.99 corresponding to the above peptide with a single iodoacetamide alkylation at C_{419} (y_9 m/z = 990.5 for the +1 ion) and one methacrylate alkylation at C_{418} (y_{10} m/z = 1179.6 for the +1 ion). The C-terminal sequence of the cysteine-containing peptide and y-ions are shown above.

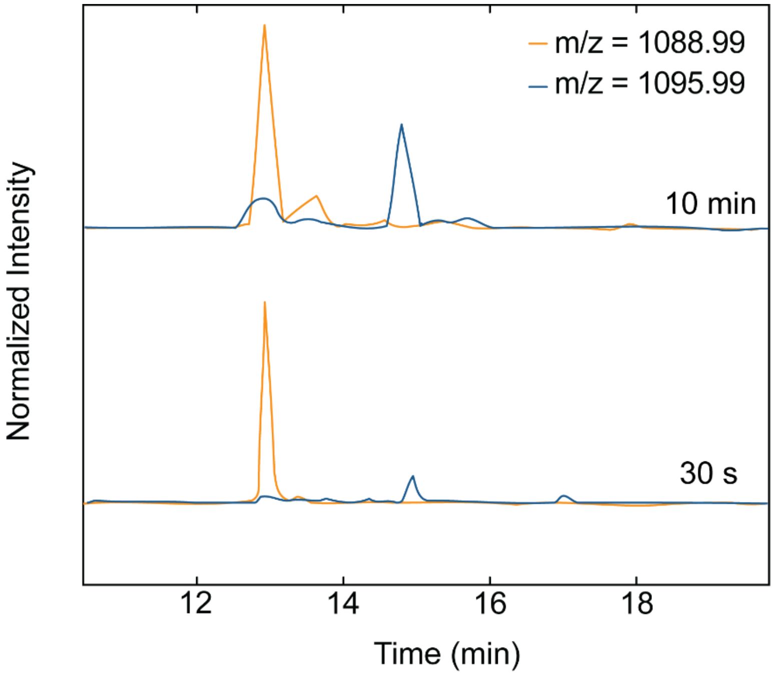

Figure S7. Data dependent LC-MS/MS chromatograms for acid-quenched reactions after and during inhibition by methacrylate. Wild type aPFL was reacted with 200 mM methacrylate and incubated for 30 s (bottom) or 10 minutes (top) at 20 °C. The reaction product was alkylated by iodoacetamide, digested with trypsin, acidified, and analyzed by LC-MS/MS. Chromatograms were generated by monitoring masses m/z of 1088.99 (orange), corresponding to the +4 ion of the NH_3^+-V_{391}SIDTSSLQYENDDLMRPDFNNDDYAIA$C_{418}C_{419}$VSPMIVG_{426}-CO_2H peptide modified with two iodoacetamide alkylations at C_{419} and C_{418} and m/z of 1095.99 (blue) corresponding to the same peptide with a single iodoacetamide alkylation at C_{419} and one methacrylate alkylation at C_{418}. Intensities were normalized for comparison.

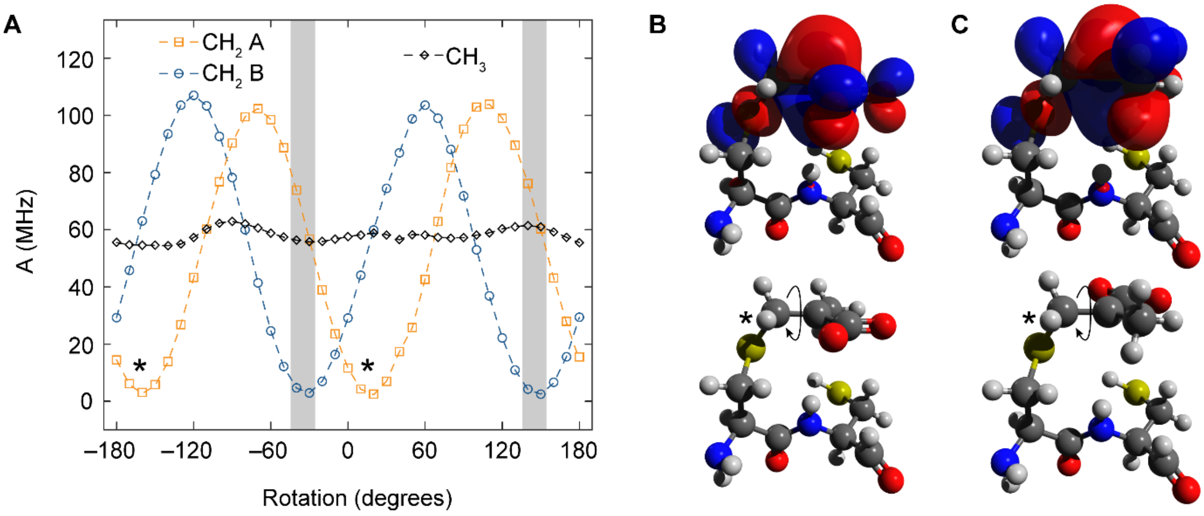

Figure S8. DFT predicted EPR HFC constants and SOMO for C2• consistent with the experimental data. **A** HFC constants at each rotamer configuration for the C3 methylene protons A (orange squares) and B (blue circles) and isotropic C4 methyl groups (black diamonds). Gray areas indicate rotamers consistent with the experimental data at approximately −35° and +145°. Asterisks indicate rotamers consistent with the experimental data, but exhibiting van der Waals clashes in the structure between the methacrylate carboxyl oxygens and the dipeptide NH. **B** Structure (bottom) and SOMO (top) of the −35° rotamer. Asterisk highlights the weakly coupled methylene ^{1}H. **C** Structure (bottom) and SOMO (top) of the +145° rotamer. Asterisk highlights the weakly coupled methylene ^{1}H. Hydrogen, white; carbon, gray; nitrogen, blue; oxygen, red; sulfur, yellow.

Table S2. DFT-calculated EPR parameters for rotamers of the C2● intermediate.

Rotation (°)	g_{iso}	A_{CH3} (MHz)	A_{CH2A} (MHz)	A_{CH2B} (MHz)
−180	2.0031	55.5	29.3	14.5
−170	2.0032	54.7	45.9	5.8
−160	2.0034	54.6	63.3	2.7
−150	2.0036	54.5	79.7	5.5
−140	2.0037	54.4	93.8	13.8
−130	2.0036	55.0	103.5	26.8
−120	2.0035	57.3	107.0	43.1
−110	2.0033	60.3	103.4	60.4
−100	2.0033	62.4	93.3	76.8
−90	2.0032	62.9	78.3	90.4
−80	2.0033	62.1	60.1	99.5
−70	2.0034	60.5	41.4	102.4
−60	2.0033	58.9	24.7	98.6
−50	2.0038	57.4	12.1	88.7
−40	2.0037	56.3	4.6	73.7
−30	2.0037	55.8	2.9	56.6
−20	2.0035	55.9	7.0	39.0
−10	2.0034	56.7	16.3	23.6
0	2.0033	57.6	29.1	11.6
10	2.0033	58.3	44.1	4.2
20	2.0033	58.9	59.9	2.32
30	2.0031	58.3	74.7	7.0
40	2.0033	56.5	86.9	17.3
50	2.0041	58.3	99.1	25.7
60	2.0041	58.2	103.7	42.7
70	2.0037	57.4	99.5	62.8
80	2.0035	57.1	88.3	81.9
90	2.0034	57.2	72.2	95.4
100	2.0033	58.1	53.1	102.9
110	2.0032	58.9	36.8	103.9
120	2.0031	60.3	22.2	99.1
130	2.0030	61.1	10.9	89.7
140	2.0029	61.5	4.1	76.1
150	2.0029	60.9	2.5	60.0
160	2.0029	59.4	6.38	43.2
170	2.0030	57.3	15.6	27.5
180	2.0031	55.5	29.3	14.5

Table S3. Composition of the EPR signal in reactions of aPFL with 200 mM methacrylate at different freeze quenching times (showed in the main text Figure 3). Relative concentrations of the C2● and G● were calculated by simulating the experimental spectra using the ***g*** tensors and hyperfine tensors in **Table S1**. Total spin concentrations were determined by comparing the double integral of the spectra to the double integral of a sample of known concentration 4-hydroxy-TEMPO.

Quench time (s)	C2● (%)	G● (%)	Radical Conc. (μM)
0	0.1 (4)	100.6 (4)	23
10	30.9 (6)	69.1 (6)	16
30	37.5 (7)	62.5 (7)	20
50	30.5 (5)	69.5 (5)	21
75	24.4 (4)	75.7 (4)	20
120	17.8 (4)	82.3 (4)	14
420	1.1 (3)	98.9 (3)	15

Table S4. Composition of the EPR signal in reactions of aPFL buffer exchanged into D_2O buffer with 200 mM methacrylate at different freeze quenching times (Figure 4). Relative concentrations of the C2• and G• were calculated by simulating the experimental spectra using the g tensors and hyperfine tensors in Table S1. Total spin concentrations were determined by comparing the double integral of the spectra to the double integral of samples of known concentration of 4-hydroxy-TEMPO•.

Quench time (s)	C2● (%)	G● (%)	Radical Conc. (µM)
0	1.7 (8)	98.3 (8)	4
30	55 (2)	44 (2)	4
90	47 (1)	52 (1)	3
150	34 (1)	65 (1)	3
225	27.4 (9)	72.6 (9)	2
360	14.0 (9)	85.6 (9)	3
720	0 (1)	100 (1)	3

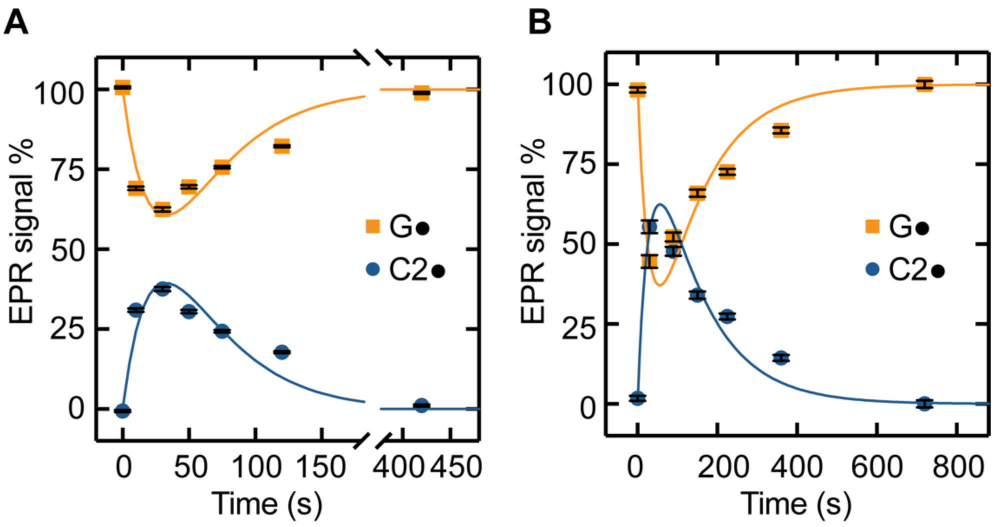

Figure S9. Global kinetic simulations for G●/C2● formation and decay. **A** Weighted EPR signals from G● (orange squares) and C2● (blue circles) in H_2O determined from spectral simulations using EasySpin and associated global fit curves (orange and blue lines). **B** Weighted EPR signals from G● (orange squares) and C2● (blue circles) in D_2O determined from spectral simulations using EasySpin and associated global fit curves (orange and blue lines). Error bars correspond to estimated error in simulation weighting.

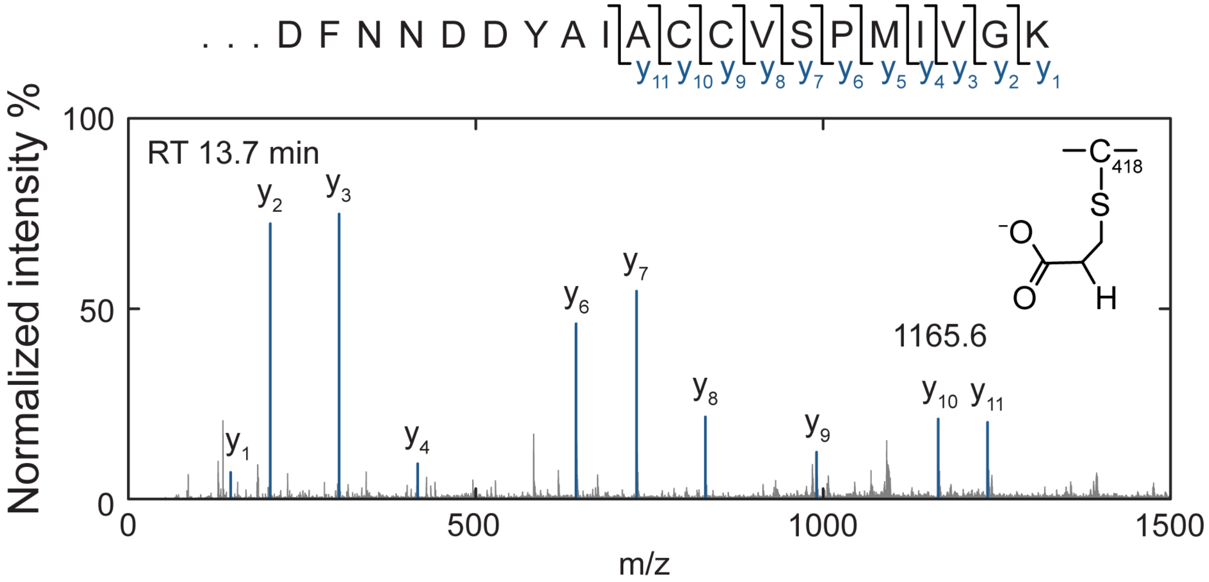

Figure S10. Peptide LC-MS/MS of aPFL inhibited with acrylate. Wild type aPFL was reacted with 10 mM acrylate and incubated for 10 minutes at 20 °C. The reaction product was alkylated by iodoacetamide, digested with trypsin, acidified, and analyzed by LC-MS/MS. Fragments of the +4 ion peptide NH_3^+-V_{391}SIDTSSLQYENDDLMRPDFNNDDYAIA$C_{418}C_{419}$VSPMIVG_{426}-CO_2H were detected with a retention time of 12.9 mins and an m/z of 1088.99 corresponding to the unreacted peptide modified with two iodoacetamide alkylations (data not shown), and a second peptide at 13.7 mins and m/z of 1095.99 corresponding to the above peptide with a single iodoacetamide alkylation at C_{419} (y_9 m/z = 990.5 for the +1 ion) and one acrylate alkylation at C_{418} (y_{10} m/z = 1165.6 for the +1 ion). Mass spectra were normalized to y_3 at 75%. The C-terminal sequence of the cysteine-containing peptide and y-ions are shown above.

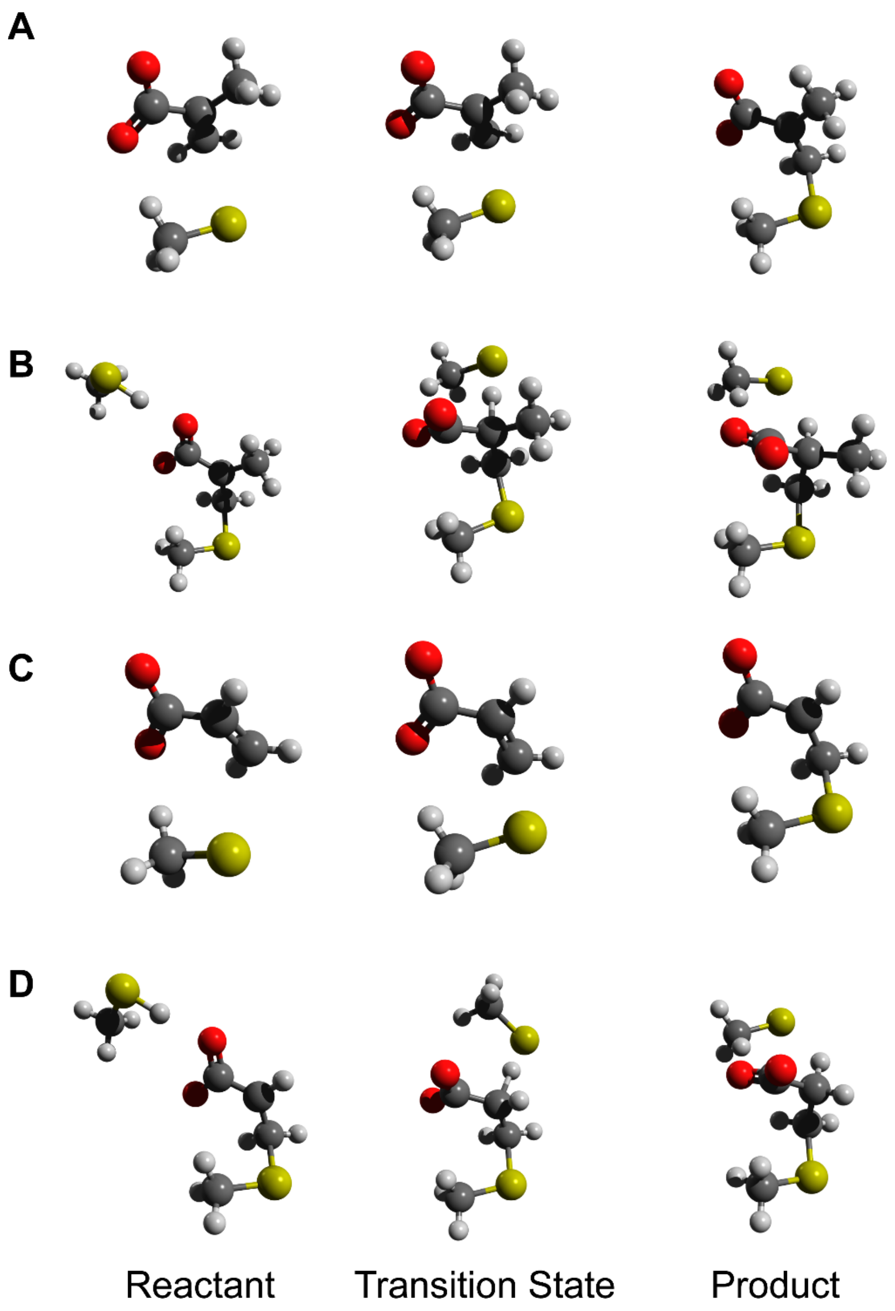

Figure S11. Geometry optimized structures for methacrylate and acrylate inhibition intermediates. **A** Acrylate step 1 (radical Michael addition). From right to left, structures correspond to the reactant state, the transition state, and the product state. Acrylate structures were generated by replacing the methyl group of methacrylate from the previously geometry optimized structures.[1] **B** Acrylate step 2 (C2● reduction by H-atom transfer). **C** Methacrylate step 1. **D** Methacrylate step 2.

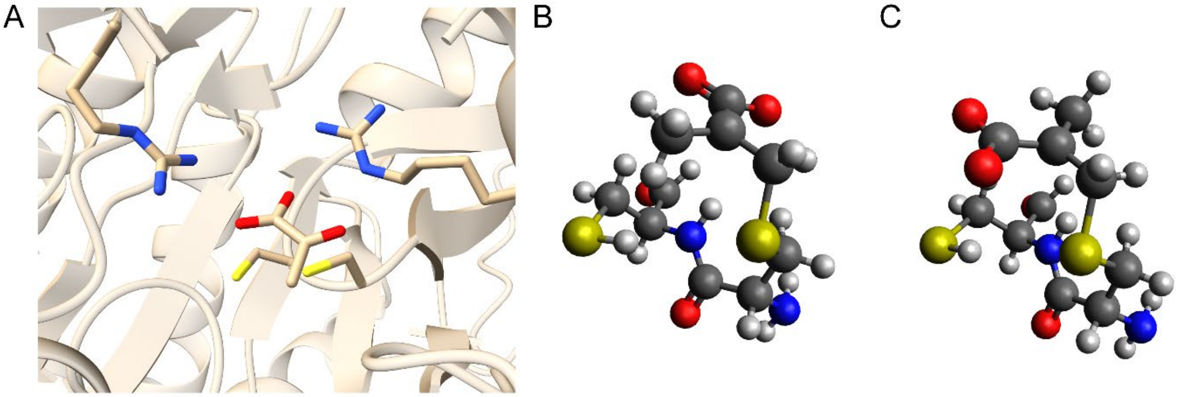

Figure S12. Structural comparison of substrate-bound PFL and C2● methacryl radical rotamers. **A** X-ray structure of pyruvate-bound *E. coli* PFL (PDB 1H18).[2] **B** DFT predicted structure of the C2● adduct in the *pro*-(*S*) configuration. **C** DFT predicted structure of the C2● adduct in the *pro*-(*R*) configuration.

References

(1) Lucas, M. de F.; Ramos, M. J. Theoretical Study of the Suicide Inhibition Mechanism of the Enzyme Pyruvate Formate Lyase by Methacrylate. *J. Am. Chem. Soc.* **2005**, *127*, 6902–6909.

(2) Becker, A.; Kabsch, W. X-Ray Structure of Pyruvate Formate-Lyase in Complex with Pyruvate and CoA: HOW THE ENZYME USES THE CYS-418 THIYL RADICAL FOR PYRUVATE CLEAVAGE. *J. Biol. Chem.* **2002**, *277*, 40036–40042.
